# An atlas of ferroptosis-induced secretomes

**DOI:** 10.1038/s41418-025-01517-4

**Published:** 2025-04-25

**Authors:** F. Isil Yapici, Eric Seidel, Alina Dahlhaus, Josephine Weber, Christina Schmidt, Adriano de Britto Chaves Filho, Ming Yang, Maria Nenchova, Emre Güngör, Jenny Stroh, Ioanna Kotouza, Julia Beck, Ali T. Abdallah, Jan-Wilm Lackmann, Christina M. Bebber, Ariadne Androulidaki, Peter Kreuzaler, Almut Schulze, Christian Frezza, Silvia von Karstedt

**Affiliations:** 1 Department of Translational Genomics, Faculty of Medicine and University Hospital Cologne, University of Cologne, Cologne, Germany; 2 Faculty of Medicine and University Hospital Cologne, CECAD Cluster of Excellence, University of Cologne, Cologne, Germany; 3 Cluster of Excellence Cellular Stress Responses in Aging-Associated Diseases (CECAD), Faculty of Medicine and University Hospital Cologne, Institute for Metabolomics in Ageing, University of Cologne, Cologne, Germany; 4 University of Cologne, Faculty of Mathematics and Natural Sciences, Institute for Genetics, Cluster of Excellence Cellular Stress Responses in Aging-Associated Diseases (CECAD), Cologne, Germany; 5 Institute for Computational Biomedicine, Faculty of Medicine, and Heidelberg University Hospital, Heidelberg University, Heidelberg, Germany; 6 Division of Tumor Metabolism and Microenvironment, German Cancer Research Center (DKFZ) and DKFZ-ZMBH Alliance, Heidelberg, Germany; 7 Institute of Medical Statistics and Computational Biology, Faculty of Medicine, University of Cologne, Cologne, Germany; 8 Center for Molecular Medicine Cologne, Faculty of Medicine and University Hospital Cologne, University of Cologne, Cologne, Germany

**Keywords:** Proteomics, Autophagy, Lipidomics

## Abstract

Cells undergoing regulated necrosis systemically communicate with the immune system via the release of protein and non-protein secretomes. Ferroptosis is a recently described iron-dependent type of regulated necrosis driven by massive lipid peroxidation. While membrane rupture occurs during ferroptosis, a comprehensive appraisal of ferroptotic secretomes and their potential biological activity has been lacking. Here, we apply a multi-omics approach to provide an atlas of ferroptosis-induced secretomes and reveal a novel function in macrophage priming. Proteins with assigned DAMP and innate immune system function, such as MIF, heat shock proteins (HSPs), and chaperones, were released from ferroptotic cells. Non-protein secretomes with assigned inflammatory function contained oxylipins as well as TCA- and methionine-cycle metabolites. Interestingly, incubation of bone marrow-derived macrophages (BMDMs) with ferroptotic supernatants induced transcriptional reprogramming consistent with priming. Indeed, exposure to ferroptotic supernatants enhanced LPS-induced cytokine production. These results define a catalog of ferroptosis-induced secretomes and identify a biological activity in macrophage priming with important implications for the fine-tuning of inflammatory processes.

## Introduction

Various types of programmed cell death can induce or modulate inflammation through the release of chemo- and cytokines as well as danger-associated molecular patterns (DAMPs) [1, 2]. Ferroptosis is a recently described type of iron-dependent regulated necrosis driven by detrimental membrane lipid peroxidation [3–5]. Herein, glutathione peroxidase 4 (GPX4) constitutively reduces accumulating lipid hydroperoxide, thereby protecting cells from ferroptosis [6, 7]. In addition to GPX4, plasma membrane-associated ferroptosis suppressor protein 1 (FSP1) generates the radical-trapping agent ubiquinol from ubiquinone, thereby providing a second line of anti-ferroptotic membrane defense in certain cancers [8–11]. Lipid peroxidation is followed by the formation of small plasma membrane pores [12, 13] and full plasma membrane rupture, which allows for the release of lactate dehydrogenase (LDH), suggesting a capacity to modulate inflammation [14, 15]. Moreover, oxidized phospholipids (oxPLs), the lipid peroxidation by-product 4-hydroxynonenal (4-HNE), high-mobility group protein B1 (HMGB1), and ATP were reported to be released from ferroptotic cells [16]. In a pancreatic cancer mouse model, ferroptotic cells were shown to release the oxidized nucleotide *8-hydroxy-2*′*-deoxyguanosine* (8-OHdG) [17]. In support of a function for ferroptosis in immune modulation, Toll-like receptor-4 (TLR4)/TIR-domain-containing adapter-inducing interferon-β (TRIF)-dependent neutrophil recruitment after heart transplantation was neutralized upon co-treatment with the ferroptosis-selective radical scavenger Ferrostatin-1 [18]. By contrast, ferroptosis-induced HMGB1 release led to activation of immune cells upon binding to *advanced glycosylation end-product specific receptor* (AGER) but not Toll-like receptor-4 (TLR4) [19]. Oxidized phospholipid, 1-steaoryl-2-15-HpETE-sn-glycero-3phosphatidylethanolamine (SAPE-OOH), can act as an eat-me signal on the surface of ferroptotic cells, which recruits macrophages via binding to *Toll-like receptor 2* (TLR2) [20]. Oxidized phosphatidylcholine was shown to inhibit the maturation and activation of bone marrow-derived dendritic cells (BMDCs) [21]. Aside from the release of established protein DAMPs and oxidized lipids, increased mRNA levels of *cyclooxygenase 2* (COX2), an enzyme that plays a vital role in generating the inflammatory mediator *prostaglandin E2* (PGE2), have been identified as a distinctive marker of ferroptosis [6]. Indeed, the knockdown of GPX4 resulted in lipid peroxidation and the release of PGE_2_ and PGF_2α_ [22].

While early studies suggested tissue ferroptosis to promote an anti-tumor inflammatory response, more recent literature has also proposed the opposite effect. Immunotherapy efficacy was partially blocked upon treatment with the ferroptosis-blocking radical scavenger Liproxstatin [23]. In line with this, mice were shown to reject tumors more efficiently when vaccinated with early ferroptotic cells [24]. CD8 T-cell-derived lFN-γ secretion cooperated with free arachidonic acid to promote ferroptosis sensitivity in tumor cells [25], and activated regulatory T cells (T_reg_) were sensitive to T_reg_-selective *Gpx4* deletion, allowing for improved anti-tumor immunity [26]_._

In contrast to these anti-tumor activities of tissue ferroptosis, ferroptosis of pathologically activated neutrophils (PMN-MDSCs) was shown to suppress anti-tumor immunity through the release of oxygenated lipids via *fatty acid transport protein 2* (FATP2) [27]. Moreover, exposure to late ferroptotic cells impaired dendritic cell (DC) cross-presentation and anti-tumor immunity [28]. Furthermore, upregulation of the scavenger receptor CD36 promoted PUFA uptake and ferroptosis of CD8 T cells, impairing anti-tumor immunity [29, 30].

Given these diverse and opposing effects on the immune response elicited, it is important to first understand the different types of potentially immunomodulatory agents released from ferroptotic cells. Importantly, an unbiased appraisal of ferroptotic secretomes has been lacking to date. Therefore, we set out to define the molecular constituents contained within secretomes of cells undergoing ferroptosis using a multi-omics approach to provide a first atlas serving as a basis for the study of immunological responses to ferroptosis.

## Results

### Characterization of proteomes released from ferroptotic cells

**Fig. 1:**
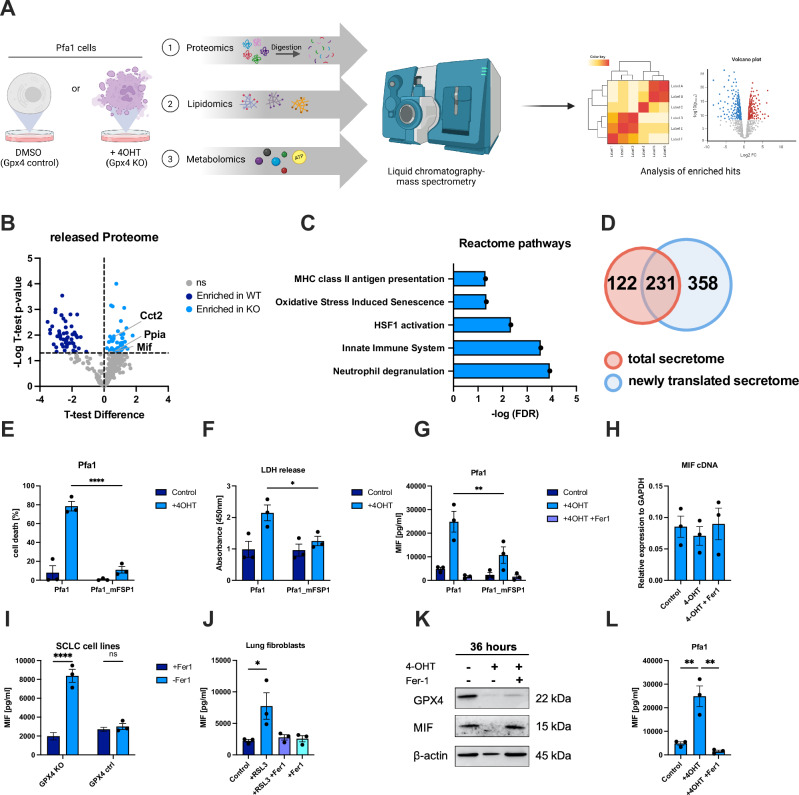
Characterization of proteomes released from ferroptotic cells. **A** Experimental setup: Pfa1 MEFs were treated with ±4OHT [1 µM] to achieve near 100% cell death and further processed for (1) proteomics (Fig. 1, 72 h), (2) lipidomics (Fig. 2, 72 h), and (3) metabolomics (Fig. 3, 48 h). **B** Volcano plot of proteins enriched in GPX4 WT (−4OHT) and KO (+4OHT) conditions is shown. *T*-test difference and corresponding −log_10_ *T*-test *p*-value are plotted for the individual hits. *p*-value < 0.05 cutoff was used to identify enriched proteins. Representative hits relevant to innate immune system pathway enrichment are highlighted within GPX4 KO supernatants. **C** Proteins significantly enriched in ferroptotic secretomes were analyzed for protein-protein interaction networks using STRING. Reactome pathways enriched within ferroptotic supernatants that are relevant to immune activation are plotted. −log_10_ of the False discovery rate (FDR) is plotted. **D** Venn diagram of the total detected secretome (red, 353 proteins in sum), newly translated secretome (blue, 589 proteins in sum), and overlapping proteins (231). **E** Parental Pfa1 or Pfa1 MEFs with stable FSP1 overexpression [9] were stained with Draq7 [100 nM] and treated with 4OHT [1 µM] for 72 h. Cells were imaged for near-infrared (NIR) count as a measure of dead cells using the IncuCyte live cell imaging system. % Cell death was normalized to confluency. **F** Supernatants from cells and treatments as in (**F**) were subjected to LDH quantification using a colorimetric assay. **G** Cells as in (**F**) were treated with 4OHT [1 µM] ± Ferrostatin-1 [1 µM] for 72 h. MIF was quantified in supernatants using ELISA. **H** Pfa1 MEFs were treated as in (**H**). qPCR-mediated quantification of MIF cDNA is shown. **I** GPX4 control or GPX4-deficient SCLC cell lines [35] were kept in the presence of Fer-1 [1 µM]. Supernatants were collected 16 h after Ferrostatin-1 withdrawal, and MIF was quantified using ELISA. **J** Primary mouse lung fibroblasts (PMLFs) were treated with RSL3 [1 µM] ± Fer-1 [1 µM] for 24 h. Supernatants were collected and subjected to MIF ELISAs. **K** Pfa1 MEFs treated ±4OHT [1 µM] ± Fer-1 [1 µM] for 36 h were subjected to Western Blot analysis of the indicated proteins. Representative blots of three independent repeats are shown. **L** Supernatant from cells, as in (**K**), was subjected to MIF ELISAs. All schemes were created with BioRender.com. Data information: **E**–**J**, **L** Graphs show data of means ± SEM of 3 independent biological replicates. One- or two-way ANOVA was used to calculate *p*-values. ns: not significant; *: *p* < 0.05; **: *p* < 0.01; ***: *p* < 0.001; ****: *p* < 0.0001. Source data are available online for this figure.

To first identify which types of proteins are released from cells undergoing ferroptosis in an unbiased approach, we made use of mouse embryonic fibroblasts with tamoxifen (4OHT)-inducible GPX4 knockout (Pfa1 MEFs) [31]. Cell death upon GPX4 deletion was entirely blocked by the ferroptosis inhibitor ferrostatin-1 (Fer-1) but not the necroptosis inhibitor nec1s or the caspase inhibitor emricasan (Fig. S1A). Moreover, Pfa1 MEFs readily accumulated lipid ROS upon induction of GPX4 knockout, further confirming the induction of ferroptotic cell death (Fig. S1B, C). Using this cellular system, we performed stable isotope labeling by amino acids in cell culture (SILAC) by culturing one part of Pfa1 MEFs in heavy and one in light isotope-containing media for a minimum of 6 passages (Fig. 1A). Lys8 and Arg10 incorporation was confirmed in these cells to reach above 90% (Fig. S1D). To keep fetal calf serum (FCS) concentrations low in supernatants for mass spectrometry analysis but circumnavigate the problem that transferrin within FCS is required for cells to undergo ferroptosis [32], we cultured cells in full FCS for 40 h after tamoxifen addition upon which we switched the media to FCS-free media supplemented with Insulin/Transferrin/Selenite + linoleic acid/bovine serum albumin (ITS+1) to further support the ability of undergoing ferroptosis for the remaining time. Under these conditions, cells also underwent ferroptosis, which was blocked by Fer-1 addition (Fig. S1E). Supernatants of Pfa1 MEFs ± 4OHT were collected after 72 h (near 100% cell death) and concentrated above a molecular weight cut-off of 10 kDa for subsequent mass spectrometry. After data processing using Perseus 1.6.15, 353 proteins were identified (Table S1), 48 of which were significantly enriched in supernatants from ferroptotic cells (Fig. 1B) and 53 were uniquely enriched in respective supernatants from live cell controls (Table S1). When analyzing the 48 proteins enriched in ferroptosis supernatants for functional association networks using STRING, we focused on immune system-related reactome pathways within 87 different reactome pathways enriched. These pathways included enrichment of MHC class II antigen presentation and innate immune system pathways, as well as oxidative-stress-induced senescence and activation of heat shock pathways (Fig. 1C). Besides the release of the total proteome, we wanted to address which proteins would be acutely or continuously translated during ferroptosis and thereby be preferentially released. To this end, we made use of heavy isotope-labeled azidohomoalanine (AHA) quantification (HILAQ) [33]. For this, cells received SILAC media including 10% FCS and were then switched into methionine-deficient media containing AHA for another 24 h. AHA is incorporated into proteins translated during this time frame and was subsequently precipitated via click chemistry beads. Addition of AHA did not impact the efficacy of ferroptosis induction (Fig. S1F and S1G). Using this method, post data processing, we detected 589 proteins (Table S2). Eef1b;Eef1b2, Eef1d, Erh, Gm9242;Gm6793, Sfpq, Snrpa, and Tpm3-rs7 were significantly enriched in ferroptotic supernatants, and 16 were uniquely enriched in supernatants from live cells (Table S2). Moreover, 231 of which were also detected in the total secretome (Fig. 1D and Table S2). Within these, there was a trend that proteins belonging to the heat shock protein family (HSPs; Hsph1, Hsp90, Hspa8, Hspa5, Hspd1, Hspa4, Hspd1), and chaperones (Cct8, Cct6a, Cct5, Cct3) were elevated in supernatants from ferroptotic cells (Tables S1 and S2). These findings might suggest enhanced endoplasmic reticulum (ER) stress, in line with a recent study identifying the ER as one of the main sites for lipid peroxidation during ferroptosis [34]. Intrigued by this, we analyzed markers of ER stress upon induction of ferroptosis. However, while tunicamycin readily induced accumulation of BiP and CHOP, indicative of ER stress, they were not affected by ferroptosis induction in our experimental system (Fig. S2). Overall, in both cases, we found an extracellular enrichment of putative DAMPs, yet bona fide chemo-cytokines known to be released from cells undergoing apoptosis or necroptosis upon TNF stimulation [1, 2] could not be detected by mass spectrometry. As chemo-cytokine abundance is often below detection thresholds in non-targeted mass spectrometry, we next used Enzyme-linked Immunosorbent Assay (ELISA) to look for chemokines described to be released under TNF-induced necroptosis, such as CXCL1 and -2. While we did detect constitutive release of CXCL1 in Pfa1 supernatants, this was reduced but not increased upon induction of GPX4 knockout (Fig. S1H). Similarly, GPX4 KO mouse small cell lung cancer (SCLC) cell lines [35] showed no release of CXCL1 (Fig. S1I).Moreover, freshly isolated primary murine lung fibroblasts (PMLFs) treated with the GPX4 inhibitor RSL3 did not show a significant release of CXCL1 and CXCL2 (Fig. S1J, K), while this was the case when they were treated to undergo apoptosis (TNF/Smac mimetic) or TNF-induced necroptosis (TNF/Smac mimetic/Emricasan) (Fig. S1L, M). Given that TNF is a known inducer of NFκB-mediated gene activation, CXCL1 release upon TNF-induced necroptosis might therefore be independent of cell death induction. Therefore, we made use of MEFs with inducible expression of Z-DNA binding protein 1 (ZBP1) [36] (ZBP1i MEFs) as a TNF-independent experimental system. Importantly, neither induction of GPX4 knockout nor ZBP1 induction led to detectable activation of NFκB (Figs. S2A and S3A). Yet, conditions triggering ZBP1-dependent necroptotic cell death (ZBP1i + Emricasan) in the absence of TNF were sufficient to induce CXCL1 and 2 release (Fig. S3B, C). Nevertheless, previous studies using ZBP1 induction have shown that inflammatory signaling can be triggered by it independently of cell death [37–39]. Notably, induction of intrinsic apoptosis (ABT737/S63845) did not do so (Fig. S3D, E). These data suggest that activating the necroptosis pathway also in the absence of TNF is sufficient for the release of these chemokines, which are lacking under the induction of ferroptotic cell death.

Interestingly, ferroptotic Pfa1 MEFs readily released lactate dehydrogenase (LDH), a stable cytoplasmic enzyme, which is commonly used to monitor extracellular release of cytoplasmic content [40], while cell death protection via overexpression of FSP1 reverted this phenotype (Fig. 1E, F). Consequently, we hypothesized that cellular ferroptosis induced through GPX4 deletion might cause the release of cytosolic DAMPs. Interestingly, macrophage migration inhibitory factor (MIF), a critical upstream mediator of innate immunity, was significantly upregulated in supernatants from ferroptotic Pfa1 MEFs and again rescued upon FSP1 overexpression (Fig. 1G). Notably, MIF mRNA was not regulated upon induction of ferroptosis (Fig. 1H). In addition, Fer-1 withdrawal from GPX4 KO but not control mouse small cell lung cancer (SCLC) cell lines [35] readily induced the release of MIF, and treatment of PMLFs with the GPX4 inhibitor RSL3 equally led to a significant MIF release from ferroptotic cells (Fig. 1I, J). A unique feature of MIF is its abundant expression and the fact that it is stored within the cytoplasm pre-made [41]. Consistently, while intracellular MIF levels were depleted upon induction of GPX4 deletion (Fig. 1K), extracellular MIF levels increased in supernatants simultaneously (Fig. 1L). Taken together, these data suggested that ferroptotic cells release pre-made proteins in a lipid ROS-dependent manner. Of note, the induction of apoptosis also slightly and the induction of TNF-induced necroptosis strongly induced the release of MIF (Fig. S1N). Together, these data provide evidence that ferroptotic protein secretomes contain putative DAMPs with innate immune activity, yet the release of bona fide chemokines as observed under necroptosis conditions is lacking.

### Ferroptosis induces the production and release of inflammatory oxylipins

**Fig. 2:**
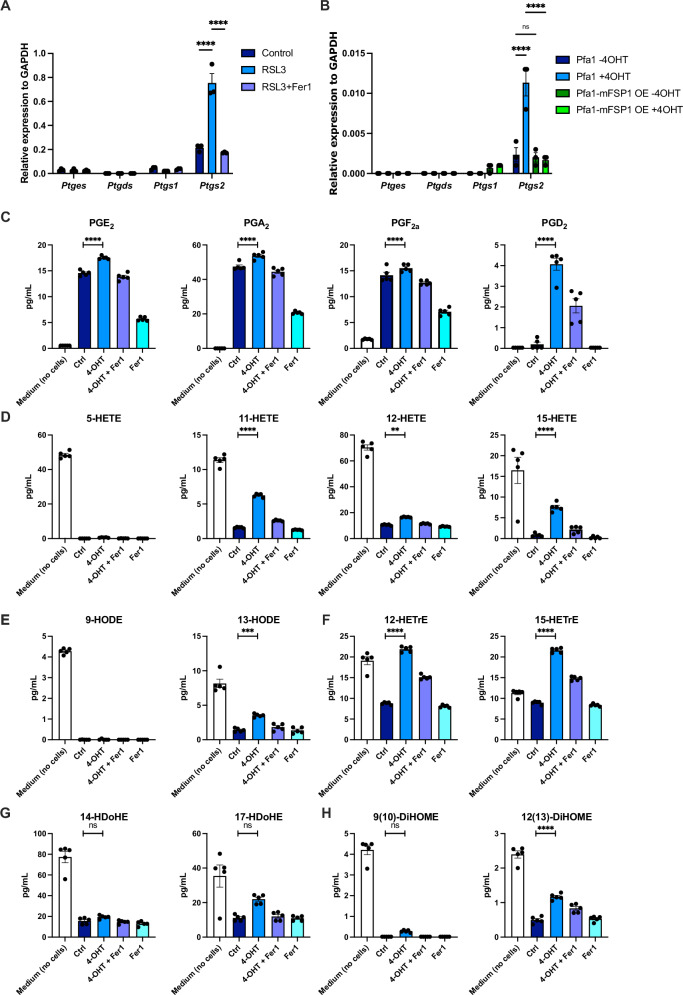
Ferroptosis induces the production and release of inflammatory oxylipins. **A** Primary mouse lung fibroblasts (PMLFs) were treated with RSL3 [1 µM] ± Ferrostatin-1 [1 µM] for 6 h. qPCR of the indicated transcripts was performed. **B** Parental Pfa1 or Pfa1 MEFs with stable mouse FSP1 overexpression [9] (mFSP1 OE) were treated ±4OHT [1 µM] for 36 h, and qPCR of the indicated transcripts was performed. **C** Pfa1 MEFs were treated - 4OHT (ctrl) or +4OHT [1 µM] ± Fer-1 [1 µM] for 72 h, supernatants and cell-free medium (*n* = 5 per condition) were collected, and concentrations of the indicated prostaglandins were quantified by mass spectrometry using standards as compared to media. **D**–**H** Supernatants from (**C**) were quantified for the indicated HETE, HODE, HETre, HDoHE, DiHOME oxylipins. Data information: **A**, **B** Graphs show data of means ± SEM of 3 independent biological replicates, and **C**–**G** 5 three independent biological replicates for lipidomics measurement. One- or two-way ANOVA was used to calculate *p*-values. ns: not significant; *: *p* < 0.05; **: *p* < 0.01; ***: *p* < 0.001; ****: *p* < 0.0001. Source data are available online for this figure.

Upregulation of prostaglandin synthetase 2 (*ptgs2/*COX2) mRNA during ferroptosis has been suggested as a combinatorial ferroptosis tissue marker [6]. COX2 catalyzes the rate-limiting step in the biosynthesis of prostaglandins, a small class of oxylipins with known immunomodulatory functions. Notably, arachidonic acid (AA) is used as an important substrate. Aside from cyclooxygenases, AA can serve as a substrate for lipoxygenases (ALOX) and cytochrome p450 (POR), all of which have been assigned functions in the promotion of ferroptosis [42–44]. Therefore, we first tested which of the corresponding mRNAs would be induced during ferroptosis. Induction of ferroptosis in PMLFs using GPX4 small molecule inhibitors RSL3 and ML210 significantly induced *Ptgs2* mRNA, which was reverted by co-treatment with Fer-1 (Figs. 2A and S4A). In support of a role for lipid ROS in ferroptosis-induced *Ptgs2* upregulation, overexpression of FSP1 in Pfa1 MEFs also reverted ferroptosis-induced *Ptgs2* (Fig. 2B).

While *Por* was also slightly induced upon ferroptosis induction using RSL3 but not ML210, none of the other enzymes were regulated upon ferroptosis induction (Fig. S4B, C). Notably, LPS-induced *Ptgs2* was not reverted by Fer-1 co-treatment (Fig. S4D, E), revealing lipid ROS as a unique means by which ferroptosis induces the prostaglandin synthesis enzymatic machinery. While *Ptgs2* induction can serve as a surrogate tissue marker for ferroptosis [6], experimental evidence for the downstream release of oxylipins during ferroptosis has been lacking. To obtain a comprehensive list of these and other oxylipins released from ferroptotic cells, we collected supernatants from control Pfa1 MEFs, Pfa1 MEFs treated with tamoxifen for 72 h (near 100% cell death) in the presence or absence of Fer-1 and treated with Fer-1 alone. These supernatants, alongside cell-free medium controls (to discern specifically released oxylipins from the ones already present at low levels in cell culture media [45]), were subjected to targeted mass spectrometry using a library of 34 oxylipins. Out of these 34 oxylipins, 19 could be detected in supernatants, 5 of which (all prostaglandins) were significantly increased over live cell and media control (specifically released) and 13 over live cell control but not media (specific changes in consumption) upon induction of ferroptosis (Table S3). Ferroptotic supernatants contained significantly increased amounts of prostaglandins (PGE2, PGA2, PDF2, PGD2) (Fig. 2C). While PGD2-derived oxylipins (15-deoxy-^Δ12,14^-PGD2 and 15-deoxy-^Δ12,14^-PGJ2) were also detected, they were not significantly increased upon ferroptosis induction (Fig. S4F, G). Co-treatment with Fer-1 reduced the amounts of prostaglandins released; however, Fer-1 alone already significantly impacted the basal release of prostaglandins from live cells. Release of PGE2 could also be confirmed upon RSL3-induced ferroptosis in freshly isolated PMLFs (Fig. S4H). Of note, PGE2 release was also detected in PMLFs undergoing extrinsic apoptosis or TNF-induced necroptosis (Fig. S4I). Notably, necroptosis induction in the absence of TNF (ZBP1i MEFs) (Fig. S3F) or induction of intrinsic apoptosis (ABT737/S63845) (Fig. S3G) did not result in significant PGE2 release, indicating that TNF but not intrinsic apoptosis or necroptosis per se result in PGE2 release. In addition, several other oxylipins were enriched in ferroptotic secretomes over live cell secretomes, a result of their decreased consumption by live cells from cell culture media upon induction of cell death (Fig. 2D–H). Notably, ferroptosis induction led to a relative enrichment of 15-hydroxyeicosatetraenoic acid (HETE) (Fig. 2D) and 13-hydroxyoctadecadienoic acid (HODE) (Fig. 2E), two AA-derived metabolites known to be generated through 15-LOX (ALOX15) activity [46]. In addition, 12,13-dihydroxy-9Z-octadecenoic acid (12,13-DiHOME), a POR-derived linoleic acid metabolite, also showed a relative increase, although not reaching the levels detected in empty media (Fig. 2H). While Lipoxin A4 (LXA4) was also detected to be constitutively consumed by live cells (Fig. S4J), its levels were elevated upon ferroptosis induction relative to live cells, indicating decreased consumption from the media. Taken together, ferroptosis induces lipid-ROS-dependent expression of *Ptgs2* and the release of oxylipins, known regulators of inflammatory processes.

### Metabolic profiles under GPX4 deletion are consistent with pentose phosphate pathway and TCA cycle activation, resulting in the enrichment of respective metabolites within ferroptotic secretomes

**Fig. 3:**
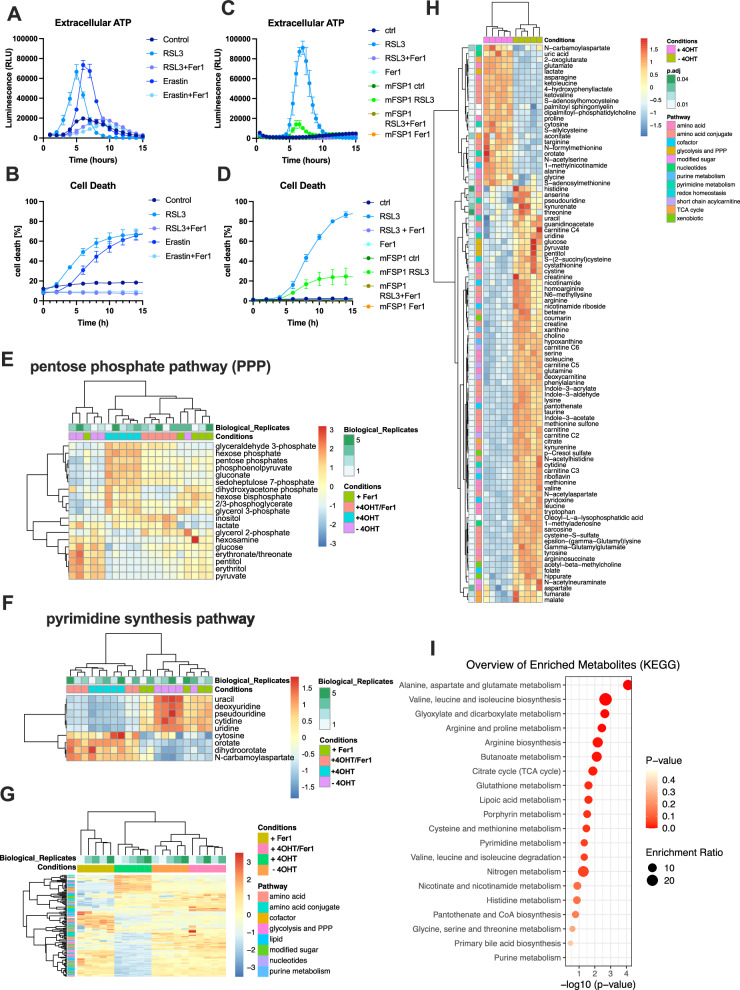
Ferroptotic cells release metabolites of active anabolism. **A** Primary mouse lung fibroblasts (PMLFs) were treated with RSL3 [1 µM] or erastin [1 µM] ± Fer-1 [1 µM] for 15 h. ATP release was measured using the RealTime-Glo™ Extracellular ATP Assay and a luminescence plate reader. Relative Luciferase Units; RLU. **B** Cells and treatments as in A were stained with Draq7 [100 nM]. Cells were imaged every 2 h for near-infrared (NIR) count as a measure of dead cells using the IncuCyte live cell imaging system. % Cell death was normalized to confluency. **C** Parental Pfa1 or Pfa1 MEFs with stable FSP1 overexpression [9] (mFSP1) were treated with RSL3 [1 µM] ± Fer-1 [1 µM], and ATP release was quantified using the RealTime-Glo™ Extracellular ATP Assay and a luminescence plate reader. Relative Luciferase Units; RLU. **D** Cells and treatments as in (**C**) were stained with Draq7 [100 nM]. Cells were imaged every 2 h for near-infrared (NIR) count as a measure of dead cells using the IncuCyte live cell imaging system. % Cell death was normalized to confluency. **E** Heatmap of pentose phosphate pathway metabolites in cell pellets 30 h after 4OHT [1 µM] stimulation of Pfa1 cells ± Fer-1 [1 µM]. Log2 fold change is shown. **F** Heatmap of pyrimidine synthesis metabolites in cell pellets 30 h after 4OHT [1 µM] stimulation of Pfa1 cells ± Fer-1 [1 µM]. Log2 fold change is shown. **G** Heatmap of all metabolites detected within supernatants of all 4 experimental conditions, 48 h after stimulation. Log2 fold distance is shown. **H** Heatmap of significantly different (*p* = 0.05) metabolites within supernatants of Pfa1 cells ± 4OHT [1 µM] for 48 h. Log2 fold distance is shown. **I** KEGG pathway enrichment of metabolites listed in (**H**) in ferroptotic supernatants (48 h + 4OHT [1 µM]) is plotted by −log^10^ *p*-value using MetaboAnalyst 6.0 [49, 50]. Data information: **A**–**D** Graphs show data of means ± SEM of 3 independent biological replicates. **E**–**H** Heatmaps show 5 independent biological replicates for metabolomics measurement. One- or two-way ANOVA was used to calculate *p*-values. ns: not significant; *: *p* < 0.05; **: *p* < 0.01; ***: *p* < 0.001; ****: *p* < 0.0001. Source data are available online for this figure.

Apoptotic cells are known to release certain nucleotides that serve as efficient DAMPs, facilitating dead cell clearance [47]. Besides these, apoptotic cells have been shown to release several other metabolites with tissue messenger activity, which can impact inflammatory responses [48]. Importantly, it has remained unexplored how intracellular metabolism is affected and what kind of small metabolites are released as a result of ferroptotic cells. Therefore, to determine the capacity of ferroptotic cells to release small molecules, we first tested for ATP release in a time kinetic. Strikingly, cells undergoing ferroptosis induced by RSL3 or erastin showed massive ATP release (Fig. 3A), preceding the onset of measurable cell death and increased lipid ROS (Figs. 3B, S5A). This release could only be measured transiently due to the low stability of free nucleotides under these conditions. Moreover, overexpression of FSP1 significantly blunted ATP release and cell death upon ferroptosis induction (Fig. 3C, D). Interestingly, despite almost similar levels of cell death achieved after 15 h induction of necroptosis using TNF/Smac mimetics/Emricasan (Fig. S5B), this only resulted in minor levels of ATP release, which was also not dependent on RIPK1 activity (Fig. S5C) and hence likely a result of TNF-induced signaling rather than RIPK1-dependent necroptotic cell death. In agreement with prior work [17], we could also confirm a significant release of the oxidized nucleotide 8-Hydroxy-2′-deoxyguanosine (8-OHdG) from ferroptotic cells (Fig. S5D). These data suggested that cells undergoing ferroptotic cell death might have a particularly high capacity to release nucleotides and small metabolites. Therefore, we next induced ferroptosis in Pfa1 MEFs through treatment with 4OHT in full FCS and performed metabolomic profiling of cell pellets at 0 h, 30 h (early, no cell death) and 48 h (late, 50% cell death), in their corresponding cell supernatants and in supernatants from 72 h stimulated cells (near 100% cell death, no pellet could be obtained) in comparison to cell-free medium. A targeted library of over 400 polar metabolites was measured by liquid chromatography mass spectrometry, 174 of which could be detected in cell pellets and 122 in supernatants. Indeed, cells undergoing ferroptosis upon GPX4 deletion (+4OHT) as compared to live cells (-4OHT) showed distinct profiles of intracellular and media metabolites (Table S4). Interestingly, upon early deletion of GPX4 in the absence of measurable cell death (30 h), cellular pellets showed a strong enrichment in purine and pyrimidine derivatives, which was attenuated, yet not entirely reverted by co-treatment with ferrostatin-1 (Fig. S5E). Notably, this early accumulation of intracellular nucleotides coincided with enrichment of pentose phosphate pathway (PPP) metabolites within cells upon GPX4 deletion (Fig. 3E). Moreover, metabolites used for pyrimidine synthesis such as orotate, dihydroorotate and cytosine were elevated in all cell pellet conditions with GPX4 deletion (Fig. 3F). Conversely, products of purine degradation including xanthine, inosine and adenosine were downregulated upon GPX4 deletion (Fig. S5F). Collectively, these data suggest activation of the PPP upon GPX4 deletion, accompanied by elevated nucleotide synthesis preceding significant levels of lipid ROS and subsequent cell death. However, the exact role of the PPP and accumulation of nucleotide derivatives during the early course of ferroptosis needs to be further elucidated in subsequent studies. In addition, we observed an early intracellular increase in TCA cycle metabolites (Fig. S5G). To determine metabolites enriched in media upon ferroptosis, we analyzed supernatants 48 h after the addition of 4OHT (50% cell death). Indeed, here supernatants from ferroptotic cells showed a clearly distinct supernatant metabolite profile which was reversed upon the addition of Fer-1 and, hence, lipid ROS dependent (Fig. 3G). Interestingly, supernatants from cells undergoing ferroptotic cell death were significantly enriched in TCA cycle, methionine cycle, purine and pyrimidine derivatives (Fig. 3H). Next, when analyzing significantly enriched metabolites from ferroptotic supernatants for metabolic Kyoto encyclopedia of genes and genomes (KEGG) pathway enrichment using MetaboAnalyst 6.0 [49, 50], we could confirm an enrichment in metabolites from the TCA cycle, methionine cycle, purine and pyrimidine synthesis as well as other pathways linked with ferroptosis protection such as the GSH pathway (Fig. 3I). While its regulation during apoptosis or necroptosis is only poorly understood, our data indicate that an early induction of the PPP and a secondary release of anabolic metabolites is triggered in cells undergoing ferroptosis.

Notably, several of the metabolites specifically enriched within ferroptotic supernatants have been assigned immune activator functions including uric acid in inducing TLR4 and TNF [51], lactate in increasing macrophage pathogen killing activity [52] and N-formylmethionine acting as a chemoattractant for neutrophils [53]. Yet, also immune-suppressive functions have been assigned to several of the metabolites enriched within ferroptotic secretomes, including dipalmitoyl-phosphatidylcholine in inhibiting TNF release from monocytes [54], lactate in suppressing response to LPS [55], glutamate in shifting macrophages towards an immune-suppressive phenotype [56], and S-adenosylmethionine in blunting LPS-induced gene expression [57]. Therefore, we next aimed to test the type of response elicited in bystander cells exposed to ferroptotic supernatants.

### Ferroptotic supernatants induce macrophage reprogramming

**Fig. 4:**
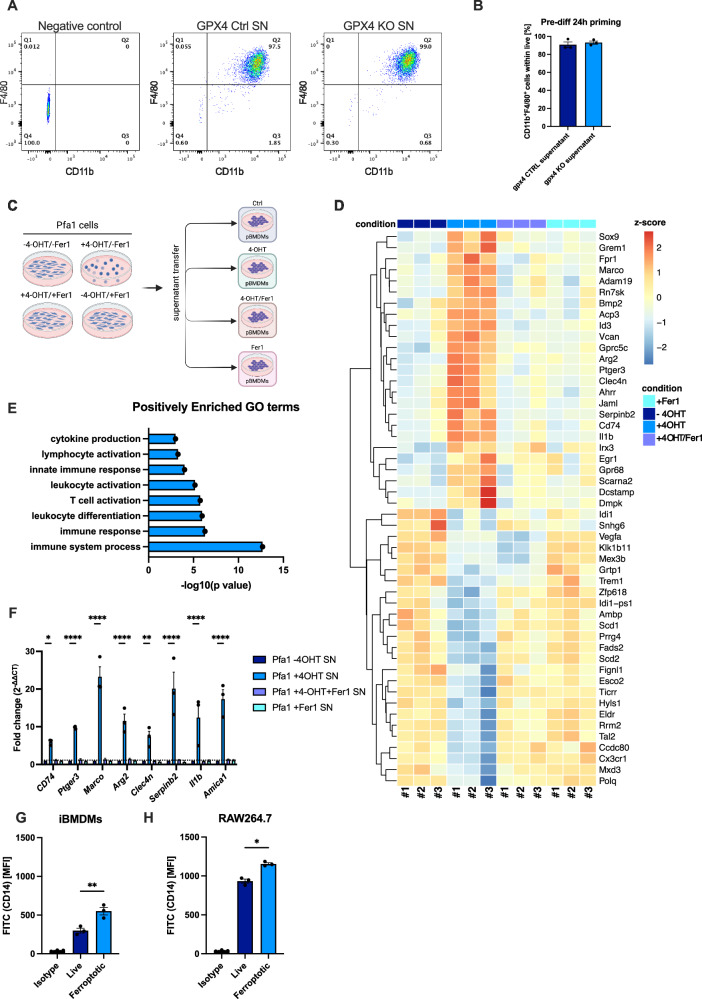
Exposure to ferroptotic supernatants induces transcriptional reprograming of macrophages. **A** pBMDMs were incubated with supernatants from GPX4 control (GPX4 ctrl SN) or GPX4 KO (GPX4 KO SN) SCLC cell lines (Bebber et al. [35]) at day 7 post-differentiation. CD11b^+^, F4/80^+^ cells were gated within live cells. Representative Flow Cytometry analysis is shown. **B** Percentage of combined CD11b^+^, F4/80^+^ pBMDMs within live cells was plotted. **C** Schematic of supernatant transfer strategy from Pfa1 to pBMDMs (*n* = 3 per condition). **D** Differentiated pBMDMs incubated for 24 h with supernatants as depicted in C were subjected to RNA sequencing. The 25 most upregulated and downregulated genes are shown. The genes are clustered row-wise by expression patterns using z-scores of normalized expression. **E** Positively enriched immune system-related GO terms in pBMDMs exposed to ferroptotic supernatants were chosen within the top 100 GO terms enriched. **F** pBMDMs were subjected to the indicated supernatants from Pfa1 MEFs for 24 h. The indicated transcripts were quantified by qPCR. **G** iBMDMs were incubated with supernatants from live (supernatant collected after 72 h from Pfa1 MEFs -4OHT) or ferroptotic Pfa1 MEFs (supernatants collected after 72 h from Pfa1 MEFs +4OHT) for 24 h. Surface expression of CD14 was quantified by antibody-based staining and flow cytometry. Mean fluorescence intensity (MFI) is shown. **H** RAW264.7 cells were incubated, stained, and analyzed as in (**G**). All schemes were created with BioRender.com. Data information: **B**, **F**, **G** Graphs and **D** heatmap show data of means ± SEM of 3 independent biological replicates. One- or two-way ANOVA was used to calculate *p*-values. ns: not significant; *: *p* < 0.05; **: *p* < 0.01; ***: *p* < 0.001; ****: *p* < 0.0001. Source data are available online for this figure.

Having identified ferroptotic cells to release a protein and non-protein secretome with putative immune-modulatory activity, we next aimed to characterize this activity utilizing a suitable cell culture-based model exploiting primary cells. To this end, we isolated primary mouse bone marrow-derived cells and differentiated them into primary F4/80^+^/CD11b^+^ bone marrow-derived macrophages (pBMDMs) (Fig. S6A, B). Next, pBMDMs were exposed to live or ferroptotic supernatants for 24 h followed by RNA sequencing (Fig. 4C). While exposure to ferroptotic supernatants did not affect pBMDM differentiation and maturation (Fig. 4A, B), interestingly, exposed pBMDMs showed a significant upregulation of *Il1b* mRNA consistent with macrophage priming [58] (Fig. 4D). Ferroptosis-exposed BMDMs also revealed an enrichment in gene ontology (GO) terms associated with immune activation (Fig. 4E, differentially expressed genes (DEG) Table S5). Of note, apart from elevated *Il1b* mRNA, which did not result in increased release of IL1b from pBMDMs as determined by ELISA (Fig. S6E, F), no direct increase in other chemo/cytokine transcripts were evident. In line with this observation, treatment of the murine macrophage cell line RAW246.4 or human THP1 cells with ferroptotic supernatants resulted in very limited production of secreted chemo-cytokines (Fig. S6C, D). Intriguingly, amongst transcripts upregulated upon ferroptotic supernatant exposure we could identify and validate a significant upregulation of CD74, a MIF receptor [59] and Ptger3, a PGE2 receptor [60] suggestive of a biological feed-forward reaction to ligands we identified to be contained within ferroptotic supernatants (Fig. 4F). Further significantly upregulated transcripts related to inflammatory processes included ADAM19, PD-L1 (CD274), non-canonical NF-kB genes NFKB2 and RELB, furthermore IFITM1, JAML -linked with acute kidney injury [61]- IRX3-linked with metabolic inflammation [62]- and PRDM1-observed in gouty arthritis [63] (Table S5). As a control, we aimed to test whether BMDMs exposed to other regulated necrosis-derived supernatants would respond similarly. To this end, we generated necroptotic supernatants from ZBP1i MEFs in the presence of caspase inhibition [36] (Fig. S6G, H) as these necroptotic supernatants would not contain TNF as a direct macrophage stimulant. Strikingly, treatment of BMDMs with these supernatants did not result in the induction of any of the genes observed upon exposure to ferroptotic supernatants (Fig. S6I). To determine whether TLRs on macrophages are required for ferroptotic DAMP sensing and transcriptional responses of pBMDMs, we made use of TLR2/4/9-deficient pBMDMs as well as MyD88- and/or TRIF-deficient pBMDMs. Interestingly, the induction of the above-mentioned mRNAs under ferroptotic supernatant exposure was completely abrogated in the absence of the selected TLRs and adapter proteins when compared to the control (Fig. S7A). Intriguingly, we also observed increased surface levels of the signal-enhancing co-receptor CD14 upon exposure to ferroptotic supernatants in various mouse macrophage lines (Fig. 4G, H), suggestive of the possibility that stimulation by TLR4 ligands might be enhanced [64]. Collectively, these data suggested that ferroptotic but not necroptotic supernatants are capable of significant and unique transcriptional reprogramming, which is initiated through TLR-mediated DAMP sensing, which nevertheless was insufficient to drive full macrophage inflammatory activation but may enhance activation via other bona fide inflammatory ligands.

### Ferroptotic secretomes promote macrophage priming

**Fig. 5:**
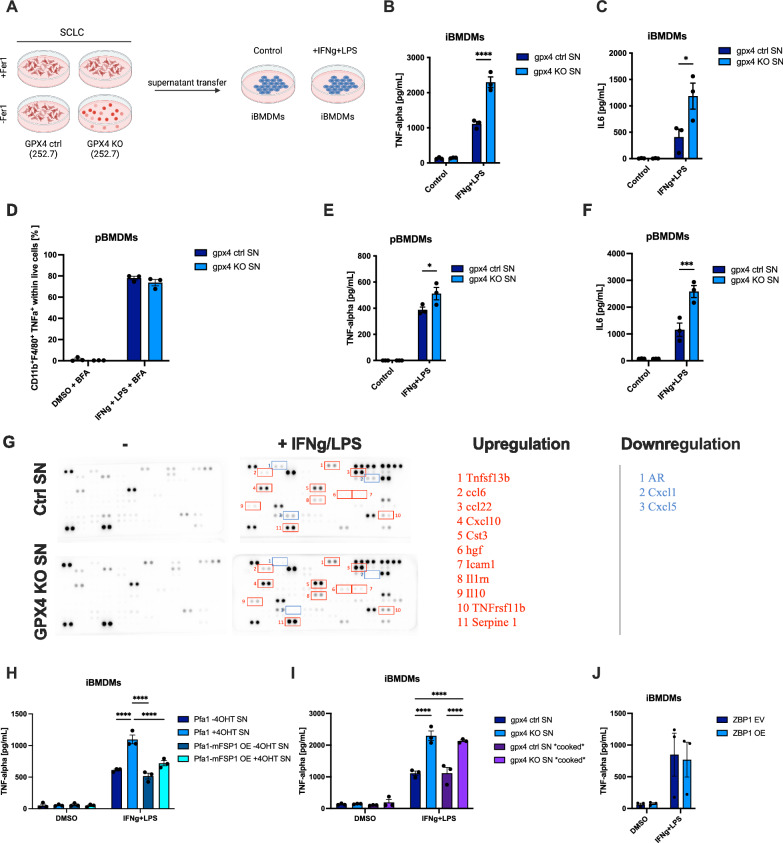
Ferroptotic supernatant exposure enhances macrophage cytokine release. **A** Schematic of the supernatant transfer strategy from GPX4 control and GPX4 KO SCLC cells 24 h after Fer-1 withdrawal to iBMDMs. After supernatant transfer iBMDMs were incubated with ctrl. or KO supernatants for 6 h with IFN gamma [25 ng/ml], after which LPS [10 ng/ml] was added for a total of 24 h. **B** iBMDMs were incubated with ctrl. (GPX4 ctrl. SN) or KO supernatants (GPX4 KO SN) from SCLC cells for 6 h with IFN gamma [25 ng/ml], after which LPS [10 ng/ml] was added for a total of 24 h. TNF-alpha was quantified using ELISA. **C** iBMDMs were incubated with ctrl. or KO supernatants from SCLC cells for 6 h with IFN gamma [25 ng/ml], after which LPS [10 ng/ml] was added for a total of 24 h. IL-6 was quantified using ELISA. **D** Differentiated pBMDMs were treated with ctrl. or KO supernatants for 6 h IFN gamma [25 ng/ml], LPS [10 ng/ml], and Brefeldin A (BFA) [5 µg/ml]. Combined CD11b^+^, F4/80^+^, TNFα^+^ cells within live cells were quantified using flow cytometry. **E** pBMDMs were incubated with ctrl. or KO supernatants for 6 h with IFN gamma [25 ng/ml], after which LPS [10 ng/ml] was added for a total of 24 h. TNF-alpha was quantified using ELISA. **F** pBMDMs were incubated with ctrl. or KO supernatants for 6 h with IFN gamma [25 ng/ml], after which LPS [10 ng/ml] was added for a total of 24 h. IL-6 was quantified using ELISA. **G** iBMDMs were incubated with ctrl. or KO supernatants for 6 h with IFN gamma [25 ng/ml], after which LPS [10 ng/ml] was added for a total of 24 h. Dot Blot analysis on the supernatants was performed using the cytokine profiler mouse XL cytokine array (R&D Systems). **H** iBMDMs were incubated with supernatants from parental Pfa1 or Pfa1 MEFs with stable FSP1 overexpression [9] (mFSP1 OE) as indicated for 6 h with IFN gamma [25 ng/ml], after which LPS [10 ng/ml] was added for a total of 24 h. TNF-alpha was quantified using ELISA. **I** iBMDMs were incubated with standard ctrl. or KO supernatants or respective boiled “cooked” supernatants as indicated for 6 h with IFN gamma [25 ng/ml], after which LPS [10 ng/ml] was added for a total of 24 h. TNF-alpha was quantified using ELISA. **J** iBMDMs were incubated with empty vector (EV) or supernatants from ZBP1 induced (ZBP1 OE) + emricasan [2.5 µM] for 6 h with IFN gamma [25 ng/ml], after which LPS [10 ng/ml] was added for a total of 24 h. IL-6 was quantified using ELISA. All schemes were created with BioRender.com. Data information: **B**–**F**, **H**–**J** Graphs show data of means ± SEM of at least 3 independent experiments. One- or two-way ANOVA was used to calculate *p*-values. ns: not significant; *: *p* < 0.05; **: *p* < 0.01; ***: *p* < 0.001; ****: *p* < 0.0001. Source data are available online for this figure.

**Fig. 6:**
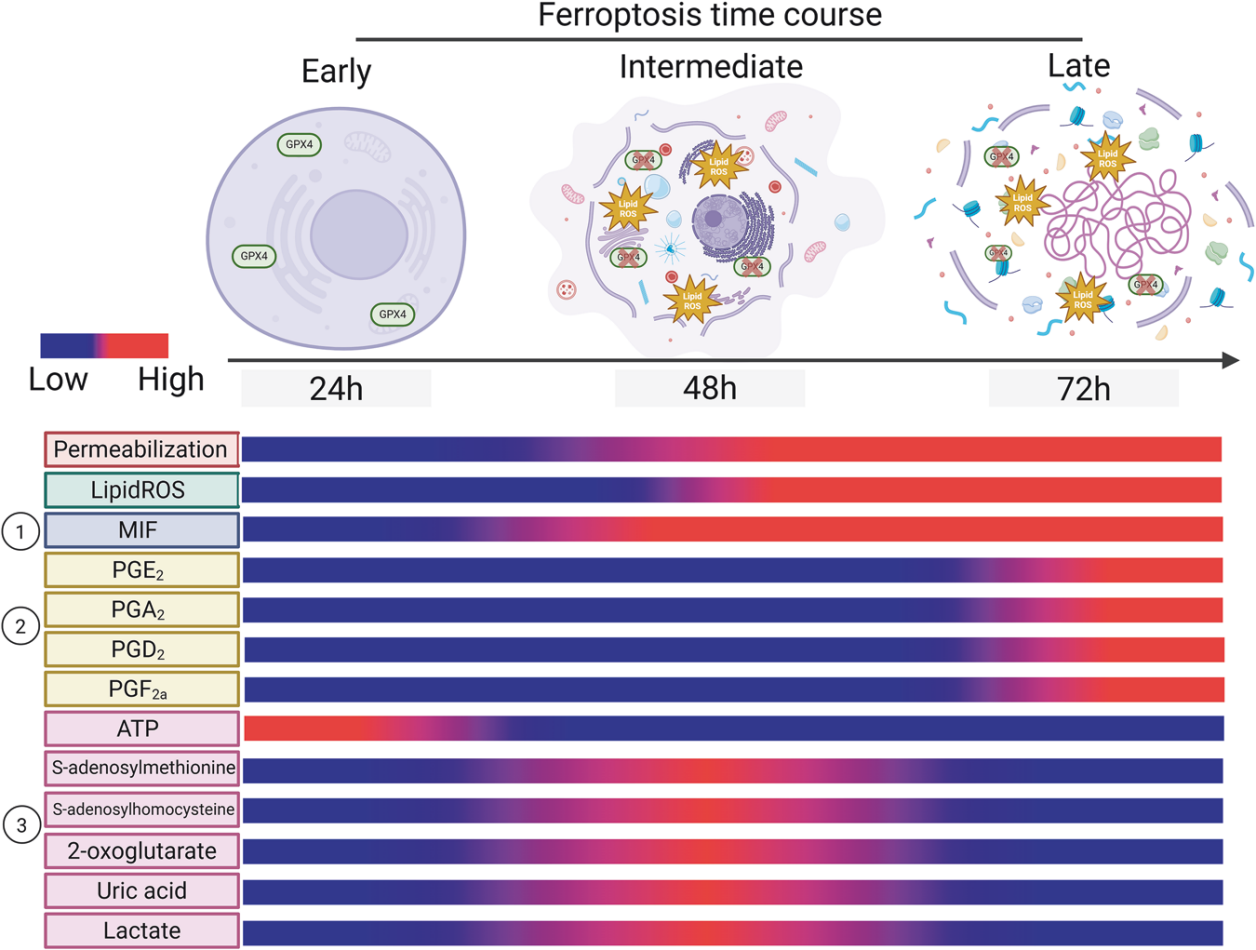
Schematic timeline of putative DAMPs released from ferroptotic cells. Timeline of cell permeabilization, lipid ROS, and the release of factors detected in (1) proteomics, (2) lipidomics, and (3) metabolomics of supernatants from ferroptotic cells. Three stages of ferroptosis can be distinguished: early ferroptosis where cells experience the release of nucleotides such as ATP; intermediate ferroptosis marking the start of lipid ROS detection accompanied by the release of MIF and metabolites involved in the TCA- and methionine cycle, and late ferroptosis with complete permeabilization of the cell with the release of LDH and prostaglandins. All schemes were created with BioRender.com.

Given the observed upregulation of surface CD14 and a transcriptional response dependent on TLRs, we next tested whether exposure to ferroptotic secretomes might enhance cytokine secretion in response to additional TLR4 stimulation via LPS [64]. To this end, we treated immortalized BMDMs (iBMDMs) [65] primed for 24 h with either control or ferroptotic supernatant with or without interferon (IFN)- gamma/LPS (Fig. 5A). Strikingly, priming with ferroptotic supernatants significantly enhanced TNF and IL-6 secretion of stimulated iBMDMs (Fig. 5B) as well as pBMDMs (Fig. S8A–C). Notably, Fer-1 treatment alone had an enhancing effect regarding IL-6 secretion from pBMDMs which may be related to its effect on basal prostaglandin synthesis (Fig. 2C). For this reason, we continued with supernatants derived from cells with a constitutive knockout of GPX4 [35] and supernatants were collected 24 h after Fer-1 withdrawal and ensuing cell death (Fig. 5A). While exposure to these ferroptotic secretomes did not change the percentage of primary TNF^+^ mature F4/80^+^/CD11b^+^ BMDMs (Fig. 5D), the amounts of TNF and IL-6 secreted were again significantly enhanced suggesting enhanced/prolonged activation of individual pBMDMs (Fig. 5E, F). In addition to TNF and IL-6, using a dot plot profiler array, we also observed a moderately increased release of Ccl22, Il1rn, Hgf, Tnfsf13b, Il10, TNFrsf11b, Icam1, Cxcl10, Cst3, ccl6, and Serpine1 from IFN gamma/LPS-treated BMDMs, which were primed with ferroptotic supernatants (Fig. 5G). Importantly, FSP1 overexpression in supernatant-producing cells was sufficient to blunt supernatant priming activity towards macrophages (Fig. 5H). Moreover, iBMDMs primed with necroptotic supernatants derived from ZBP1i MEFs did not show enhanced TNF or IL-6 secretion as compared to vector control supernatants upon stimulation (Fig. 5J, S8E). To narrow down whether the “priming substance” within ferroptotic supernatants was part of the free soluble protein or residual secretome, we boiled ferroptotic supernatants to denature soluble protein content. Of note, proteins residing within extracellular vesicles (EVs) are known to withstand such treatment. Interestingly, boiled supernatants from ferroptotic cells retained their priming activity, strongly suggesting the residual fraction released from ferroptotic cells to mediate this effect (Fig. 5I, S8D). Notably, a recent study comparing various modes of regulated cell death also characterized the release of extracellular vesicles (EVs) upon ferroptosis [66]. Given that EVs can withstand heating and boiling, supernatants retained their priming activity, we next tested whether EVs released from ferroptotic cells might be involved in macrophage priming. To this end, we repeated the priming/exposure experiments in iBMDMs using EV-depleted vs. complete supernatants of control vs. ferroptotic cells. Interestingly, exposure to EV-depleted ferroptotic supernatant partially lost its priming activity, suggesting that EVs contribute to ferroptosis-associated macrophage priming (Fig. S8F, G). Collectively, these data provide a first atlas of putative DAMPs released from ferroptotic cells, an overview of their timing (Fig. 6), and demonstrate that ferroptotic secretomes, in particular the EV-containing fraction, show a unique capacity to prime macrophages for activation, which is not shared by necroptotic supernatants.

## Discussion

Tissue ferroptosis is increasingly recognized to be involved in various inflammatory conditions [67], yet the class of agents released from ferroptotic cells has remained elusive. In a multi-omics approach, we have provided a first comprehensive and unbiased view on proteins, oxylipins, and metabolites released from ferroptotic cells and comparatively benchmarked some key findings against necroptotic supernatants. Interestingly, while several common proteins were released from ferroptotic and necroptotic cells, including MIF, ZBP1-induced necroptotic cells released typical chemokines such as CXCL1 and -2 [68], suggesting that engaging the necroptosis machinery, unlike ferroptosis is capable of triggering the release of inflammatory chemo-/cytokines even in the absence of the death ligand TNF. Yet chemo-cytokines released from necroptotic cells might be a result of cell death-independent gene induction via RIPK3 kinase activity- and MLKL-dependent inflammatory signaling [37–39]. Additionally, mass spectrometry after enriching for actively translated proteins using HILAQ did not reveal chemo- or cytokines in secretomes from cells undergoing ferroptosis. This lack of newly translated chemo-cytokines might be explained by the lack of RIPK3 activation during ferroptosis as opposed to necroptosis [3], as well as possible loss of secretory translation as a result of ER membrane peroxidation [34]. In addition, given the ATP efflux and high mitochondrial anabolic activity we observed in cells undergoing ferroptosis and the high ATP expenditure of translation [69], it is possible that translation as a whole is limited to maintenance of the core minimal translational machinery in these cells. Notably, due to different abundance of peptides, some protein classes might nevertheless not be detected without their specific enrichment or prefractionation in mass spectrometry. Especially proteins with low molecular weights, including most cytokines, provide less peptides. Indeed, although CXCL1 and -2 were not detected within our proteomic analysis, ELISA quantification confirmed that they were indeed constitutively released, but, importantly, not increased upon induction of ferroptosis. Interestingly, translated ferroptotic secretomes contained many chaperones, factors involved in ER stress response [70]. Indeed, protein folding within the ER is very redox sensitive, and already slight changes in redox homeostasis can cause ER stress and an unfolded-protein response (UPR) [71]. Recently, the ER has been identified as one of the main sites for lipid peroxidation during ferroptosis [34]. Interestingly, the specific protein content of EVs from ferroptotic cells has been suggested to be associated with ER stress [66], which might result from lipid peroxidation at mitochondria and the ER. Yet, in our experimental setting, we could not detect the accumulation of bona fide ER stress markers. However, this does not exclude the possibility of local ER stress occurring transiently during ferroptosis, hence remaining below the level of detection for the ER stress markers investigated while contributing to the observations reported in this study. Moreover, extracellular chaperones are also known modulators of the inflammatory response, albeit in a context-dependent manner [72]. Activation of heat shock pathways upon ferroptosis induction also falls in line with previous studies identifying several members of HSPs modulating ferroptosis via reducing iron uptake and/or increasing lipid peroxidation through GPX4 degradation [73, 74]. The observed enrichment in oxidative-stress-induced senescence profiles could be an indirect result of changes in iron metabolism [75] and the depletion of glutathione [76].

Notably, glyceraldehyde-3-phosphate dehydrogenase (GAPDH), lactoylglutathione lyase (GLO1), and triose phosphate isomerase (TPI1) were all significantly released from ferroptotic cells (Table S1). Importantly, GAPDH and TPI1 are critical enzymes in catalyzing glycolysis, and GLO1 plays an important role in detoxifying glycolysis byproducts [77]. Indeed, ferroptotic cells also released increased amounts of lactate, suggestive of increased rates of glycolysis (Fig. 3H). Together, these data suggest that ferroptotic cells might undergo enhanced glycolysis to maintain basal levels of energy homeostasis, resulting in a protein secretome reflecting this metabolic state.

Aside from the released protein fraction, we cataloged several oxylipins with inflammatory potential, most importantly, prostaglandins. Yet, interestingly, Ferrostatin-1 significantly lowered basal release of prostaglandins, suggesting lipid ROS to be involved in their constitutive production. Indeed, in line with earlier reports [6], induction of *PTGS2*, the gene encoding for cyclooxygenase 2, which synthesizes prostaglandin E_2_ (PGE_2_), was also dependent on lipid ROS (Fig. 2A, B). While lipid ROS-dependency of *PTGS2* and PGE_2_ induction was unique to ferroptosis, PGE_2_ release from dying cells seems to be a common immune inhibitory event in necrotic and non-necrotic types of cell death [78, 79]. Although necroptotic cells were shown to also release PGE_2_, our data suggest that this may very much depend on which route necroptosis was initiated (TNF-dependent or -independent).

Importantly, directly comparing ATP release from cells undergoing ferroptosis with cells undergoing TNF-induced necroptosis, we found that a strong release of ATP is a characteristic feature of cells dying in response to GPX4 inhibition. Of note, this level of release was significantly blunted with overexpression of FSP1, suggesting that limiting lipid peroxidation via FSP1-enhanced production of radical-trapping ubiquinol [8, 9] is also sufficient to revert this feature. While we did observe ATP release upon GPX4 inhibition within hours of stimulation, in 48 h supernatants of GPX4-depleted cells, this was not detectable anymore, possibly due to its limited stability. Interestingly, GPX4-depleted cell pellets showed a strong accumulation of intracellular nucleotides along with increased intermediates of nucleotide synthesis and the PPP preceding cell death. Notably, this increase in intracellular nucleotides was only very mildly reverted by Fer-1 treatment, which is not surprising given that at this early time point, lipid ROS was not significantly elevated yet. Indeed, prior studies in the context of generic oxidative stress report a ROS-dependent drop in NADPH pools to cause an induction of the PPP and ensuing nucleotide synthesis and NADPH generation [80]. Hence, it is tempting to speculate that, also in the context of GPX4 deletion and the observed loss of GSH, general ROS might be driving the induction of the PPP rather than lipid ROS.

One of the phenotypic characteristics of ferroptosis described is mitochondrial fragmentation [3]. Mitochondrial fragmentation triggered as a result of genetic OXPHOS deficiency was observed to come with induction of the integrated stress response (ISR), which in turn suppressed ferroptosis [81]. Interestingly, activation of the ISR was shown to upregulate *Ptgs2* [82], which is one of the marker genes upregulated in cells undergoing ferroptosis [6]. Based on these findings, it is tempting to speculate whether, during ferroptosis, an ISR might be triggered as a negative feedback loop in surviving cell populations, a hypothesis which might explain the enrichment in the core translation machinery observed in the released proteome.

Another interesting observation we made was the fact that metabolites released from ferroptotic cells strongly overlapped with metabolites required in actively proliferating cells. For active proliferation, cells undergo significant metabolic shifts, many of which are required to sustain large-scale nucleotide biosynthesis. Glutaminolysis is engaged as a source for nitrogen units, with the remaining carbons usually entering the TCA cycle via glutamate and α-ketoglutarate and then either being fully cycled or shuttled towards aspartate. Interestingly, the majority of intermediates on this metabolic axis can be found in ferroptotic supernatants. Equally, methionine-cycle metabolites, found to be enriched in ferroptotic supernatants, are known to be important for proliferating cells [83, 84]. In agreement with this hypothesis, several methionine-cycle intermediates as well as products, such as S-adenosylmethionine (SAM), S-adenosylhomocysteine (SAH), and 1-methylnicotinamide (MNA), are found to be abundant in ferroptotic supernatant. Apart from that, SAM treatment of BMDMs enhanced LPS-induced pro-IL-1ß expression by promoting one-carbon flux [85], and SAM derived from cell corpses killed by UV exposure was shown to promote efferocytosis-induced macrophage activation [86]. While this study did not further resolve the type of cell death induced by UV irradiation, another study revealed that UVB exposure triggered ferroptosis in keratinocytes and skin inflammation [87].

Another heat-stable component [88] within ferroptotic supernatants is EVs. Notably, depletion of EVs from ferroptotic supernatant resulted in decreased priming of iBMDMs (Fig. S8F, G). This suggests that EVs in the heat-inactivated fractions likely remained functional. Interestingly, in a cell culture model of pre-eclampsia, EVs seem to serve as DAMP carriers upon induction of ER stress [89]. Moreover, exosomes, a subclass of EVs, derived from breast cancer cells undergoing ferroptosis, have been shown to directly interfere with macrophage polarization, thereby suppressing the cancer cell line’s migratory and invasive potential [90]. EVs can carry various types of proteins as cargo [91]. A recent proteomics study of EVs released from live as compared to necroptotic, apoptotic, and ferroptotic cells reported that the cargo of these EVs consisted of proteins implicated in RNA splicing, processing, localization, and export [66]. Of note, acetone precipitation preceding our protein mass spectrometrical analysis of secretomes breaks EVs and therefore includes their content in the analysis. Indeed, proteins involved in splicing and translation were also discovered during our proteomic assessment of newly translated proteins during ferroptosis (Fig. 1, Table S2), directly confirming the upregulation of translation elongation factors Eef1b and Eef1d, as well as splicing factors Sfpq and Snrpa reported in the previous study. Aside from protein cargo, EVs are known to also contain non-protein content, including metabolites and lipids within their membranes. For instance, EVs released by cellular in-vitro models contain a broad portfolio of different lipids [92], and macrophage priming of iPSC-derived EVs is mediated by their glycosphingolipid components [93]. Interestingly, TLR4 has been demonstrated to be activated by EV-borne oxidized lipids in a mechanism similar to LPS, thereby functioning as a sensor for oxidative stress [94]. Given that lipid peroxidation is a hallmark of ferroptosis, these insights might hint at the possibility that these effects may be caused by the oxidized lipid fraction of EVs secreted during ferroptosis. Building on these combined findings and further evidence showing that EVs derived from human and murine erythrocytes may prime BMDMs and enhance secretion of TNF and IL-6, likely by increasing activity of the TLR4-MyD88 axis [95], it was striking to note that deleting TLR2/4/9 or the adapter proteins MyD88 and TRIF in pBMDMs abolished the priming effects of ferroptotic supernatants (Fig. S7A). Due to its unique feature of signaling through both MyD88 and TRIF, as well as its affinity to LPS used in the priming experiments, we propose that at least some of the priming activity of ferroptosis-associated EVs is mediated via TLR4/MyD88/TRIF signaling. However, while EV-depletion attenuated the release of TNF and IL-6 from primed BMDMs, it did not abrogate ferroptosis supernatant priming entirely. This may result from technical issues such as incomplete removal of EVs, but may also suggest that the observed effects might be caused by multiple players, which perhaps may engage in synergistic effects. Notably, necroptotic supernatants obtained from ZBP1i MEFs were not capable of enhancing LPS-induced cytokine secretion and also showed very minor ATP release. Yet, the relatively limited activation of macrophages observed without the addition of LPS might be caused by a unique mixture of pro- and anti-inflammatory modulators contained within ferroptotic supernatants, where TLR2/4/9 and the adapter proteins MyD88 and TRIF also play a role, as their presence was vital for the priming effect. Given that any one of the metabolites, oxylipins, but also non-protein mediators not assayed for in this study, might mediate these priming effects on macrophages, extensive future studies will be required to narrow down the classes of these modulators.

In conclusion, our study provides a first catalog of ferroptotic secretomes and compares exemplary factors with their release from necroptotic cells. While we find many features to be shared profiles of release from cells undergoing regulated necrosis, we identify (i) absence of CXCL1/2 as a feature distinguishing ferroptotic from necroptotic secretomes, (ii) significant early ATP and metabolite release as a highly characteristic feature of ferroptosis, supporting the current idea of ferroptosis as a metabolic type of cell death [96] and (iii) a unique BMDM priming activity within the ferroptosis-associated EV-enriched secretome mediated through TLR DAMP sensing. Collectively, our data offer a first basis for investigating the complexity and possibly cell-type specificity of ferroptosis-elicited immune responses in the years to come.

## Methods

### Reagents and tools table (see Table 1)

#### Data and code availability

Proteomics, metabolomics, lipidomics, and RNA-seq datasets generated within this study have been deposited at public repositories (please refer to the reagents and tools table) and are publicly available upon publication. All other original data published within this study are available from the corresponding author upon request. This paper does not report original code. Any additional information required to reanalyze the data reported in this work paper is available from the lead contact upon request.

### Animals for tissue harvest

Mice from the C57BL/6N strain were obtained from in-house breeding at the CECAD in vivo research facility at the Cologne University Medical Center, Cologne, Germany, and housed in compliance with animal welfare regulations according to the local animal welfare authority (Landesamt für Natur, Umwelt und Verbraucherschutz Nordrhein-Westfalen, Germany) under specific-pathogen-free conditions with food and water ad libitum and a regular 12-h light-dark cycle. For tissue harvest, animals were handled and sacrificed in compliance with Directive 2010/63/EU. Primary murine bone marrow-derived macrophages (pBMDMs) [97] and primary lung fibroblasts (PMLFs) [98] were generated as previously described.

### Cells

Murine SCLC cell lines and primary BMDMs were cultured in RPMI; native lung fibroblasts, CL13 iBMDMs, Pfa1, and Pfa1-mFSP1 OE MEFs were cultured in DMEM GlutaMAX™. All cells were kept at 37 °C with 5% CO2, and all media were supplemented with 10% FCS and 1% P/S. All cell lines were tested for mycoplasma at regular intervals (mycoplasma barcodes, Eurofins Genomics).

### Preparation of extracellular vesicle (EV)-depleted supernatants

Supernatants from ferroptotic cells were depleted from extracellular vesicles as previously described [99]. Pfa1 MEFs were seeded on a 15 cm dish (200,000 cells per plate), treated with 1 µM tamoxifen, and supernatants were harvested after 72 h of incubation. Supernatants were centrifuged to remove floating cells and debris (1250 rpm, 5 min, 4 °C) and subsequently subjected to ultracentrifugation for 18 h at 134,434 × *g* and 4 °C using an Optima L-80XP ultracentrifuge equipped with a SW41 rotor (both from Beckmann Coulter). Afterwards, supernatants were carefully collected, leaving 1 mL of liquid above the visible pellet. Pellets were resuspended in the remaining amount of medium. Both pellets and supernatants were stored at −80 °C until further use. Both fractions were tested in a western blot for the marker TSG101, indicative of EV-enrichment in the pellet fraction.

### Total proteomics in ferroptotic supernatants of Pfa1 cells using SILAC-LC-MS/MS

Pfa1 MEFs were labeled with heavy Arg10 and Lys8 using SILAC DMEM with 1% glutamine, 10% FBS, and 1% PS for 6 passages, and isotope integration was confirmed via mass spectrometry as described below. For ferroptotic supernatant harvest, 300,000 cells were seeded on 15 cm dishes in labeled medium in the presence of 1 µM tamoxifen or in unlabeled medium in the presence of DMSO (triplicates for each condition). After 40 h, medium was replaced with heavy or unlabeled SILAC medium containing 0% FCS and 1% ITS + 1. 72 h later, supernatants were collected, cleared by centrifugation (3 min; 300 g: 4 °C) and concentrated using Amicon Ultra centrifugal filters (Sigma Aldrich) at a 10 kDa cutoff for 25 min at 3200 × *g* and 4 °C followed by another 10 min at 3200 × *g* upon adding 10 mL ice-cold PBS. Proteins were precipitated from filtrates using the 4-fold volume of ice-cold acetone for 15 min at −80 °C followed by overnight incubation at −20 °C and centrifugation for 15 min at 16,000 × *g* and 4 °C. Air-dried pellets were then lysed in 100 µL lysis buffer (8 M Urea in 50 mM TEAB containing protease inhibitor cocktail), and nucleic assays were removed using 50 U Benzonase HC (Merck Millipore) at 37 °C for 30 min followed by centrifugation at 20,000 × *g* for 15 min. 50 µg of pooled protein (25 µg unlabeled and labeled, respectively) was then digested by subsequent treatment with 5 mM DTT (25 min, 1 h), 40 mM chloracetamide (30 min at rt), LysC endopeptidase (enzyme: substrate ratio of 1:75; 4 h at 25 °C) and trypsin (enzyme: substrate ratio of 1:75; overnight at 25 °C). The next day, samples were acidified by adding formic acid to a final concentration of 1%. Samples were cleared by centrifugation (full speed for 5 min) and loaded onto a stage tip column (5 min at 2600 rpm), followed by washing with 0.1% formic acid and 0.1% formic acid in 80% acetonitrile and air-drying.

All samples were analyzed by the CECAD Proteomics Facility on a Q-Exactive Plus Orbitrap (Thermo Scientific) mass spectrometer that was coupled to an EASY nLC (Thermo Scientific). Peptides were loaded with solvent A (0.1% formic acid in water) onto an in-house packed analytical column (50 cm, 75 µm inner diameter, filled with 2.7 µm Poroshell EC120 C18, Agilent). Peptides were chromatographically separated at a constant flow rate of 250 nL/min using the following gradient: 3–5% solvent B (0.1% formic acid in 80% acetonitrile) within 1.0 min, 5–30% solvent B within 121.0 min, 30–40% solvent B within 19.0 min, 40–95% solvent B within 1.0 min, followed by washing and column equilibration. The mass spectrometer was operated in data-dependent acquisition mode. The MS1 survey scan was acquired from 300 to 1750 m/z at a resolution of 70,000. The top 10 most abundant peptides were isolated within a 1.8 Th window and subjected to HCD fragmentation at a normalized collision energy of 27%. The AGC target was set to 5e5 charges, allowing a maximum injection time of 55 ms. Product ions were detected in the Orbitrap at a resolution of 17,500. Precursors were dynamically excluded for 25.0 s. All mass spectrometric raw data were processed with MaxQuant 2.0.3 [100] using default parameters. Briefly, MS2 spectra were searched against the canonical murine Uniprot reference proteome (UP000000589, downloaded on 26.08.2020), including a list of common contaminants. False discovery rates on protein and PSM levels were estimated by the target-decoy approach to 1% (Protein FDR) and 1% (PSM FDR), respectively. The minimal peptide length was set to 7 amino acids, and carbamidomethylation at cysteine residues was considered as a fixed modification. Oxidation (M) and Acetyl (Protein N-term) were included as variable modifications. Multiplicity was set to 2, and Arg10 as well as Lys8 were defined as labels for the heavy channel, and the re-quantify option was enabled. The match-between runs option was enabled between replicates in each sample group. LFQ quantification was enabled using default settings. Final data analysis was performed in Perseus 1.6.15 [101] based on normalized ratios between heavy and light channels.

### Proteomics of newly translated proteins in ferroptotic supernatants of Pfa1 cells using click chemistry-LC-MS/MS

Click chemistry-LC-MS/MS of newly translated proteins was done as described previously with several modifications [102]. 1 million Pfa1 cells were seeded into 175 cm^2^ in the absence or presence of 1 µM tamoxifen. After 24 h, cells were washed with pre-warmed PBS, and intracellular amino acids were subsequently for 30 min using amino-acid free SILAC medium supplemented with 1% penicillin streptomycin, dialyzed FBS, 100 mg proline, 160 mM leucine, and 1%pyruvate. Afterwards, medium was replaced with SILAC medium containing 100 µM AHA, adding intermediate SILAC amino acids (0,84 μl/ml 13C6 -Arg; 1.46 μl/ml d4 -Lys) to control cells, while heavy SILAC amino acids (0.84 μl/ml 13C6 15N4 -Arg; 1.46 μl/ml 13C6 15N2 -Lys) were fed to tamoxifen-treated cells. After 24 h, supernatants were collected and centrifuged (12000 rpm for 5 min). Protease inhibitor was added, and supernatants were concentrated using Amicon Ultra centrifugal filters (Sigma Aldrich) at a 10 kDa cutoff. Click chemistry was done from concentrated supernatants using the Click Chemistry Capture Kit according to the manufacturer’s instructions. Afterwards, resin was incubated with 500 µL SDS wash buffer (1% SDS) and 5 µL 1 M DTT at 70 °C for 15 min followed by centrifugation for 5 min at 1000 × *g*, washed with 40 mM chloroacetamide for 30 min, and subsequently loaded onto a spin column. Columns were washed with 8 M Urea/100 mM Tris-HCl (pH 8), 20% Isopropanol, and 20% Acetonitrile (ACN). Then, proteins were digested using 500 μL digestion buffer (50 mM TEAB, 2 mM CaCl2, 10% Acetonitrile) followed by treatment with 1 μg/ml trypsin and 0.5 μg/ml LysC at 37 °C overnight in a shaker at 800 rpm. Peptides were then loaded onto stage tips and further prepared for mass spectrometry as described above.

All samples were analyzed as described above, with minor changes to the settings: Peptides were chromatographically separated using the following gradient: initial 3% solvent B (0.1% formic acid in 80% acetonitrile), 3–5% B within 1.0 min, 5–30% solvent B within 40.0 min, 30–50% solvent B within 8.0 min, 50–95% solvent B within 1.0 min. The mass spectrometer was operated in data-dependent acquisition mode. The MS1 survey scan was acquired from 300 to 1750 m/z at a resolution of 70,000 and 20 ms maximum injection time. After HCD fragmentation, precursors were dynamically excluded for 10.0 s during Orbitrap detection. Mass spectrometric raw data were processed with MaxQuant (version 1.5.3.8) [100] using default parameters against the murine Uniprot database (downloaded 16.01.2019) with the re-quantify option activated. Multiplicity was set to three, and Lys4/Arg6 (medium labels), as well as Lys8/Arg10 (heavy labels), were defined. Follow-up analysis was done in Perseus 1.6.15 [101]. Protein groups were filtered for potential contaminants and insecure identifications. Remaining IDs were filtered for data completeness in at least one group.

**Table 1:** Resource table.

REAGENT or RESOURCE	SOURCE	IDENTIFIER
**Antibodies**		
Anti-mouse CD16/32 Antibody	Biolegend	Cat# 101302 RRID: AB_312801
APC anti-mouse/human CD11b Antibody	Biolegend	Cat# 101212 RRID: AB_312795
Brilliant Violet 421™ anti-mouse TNF-α Antibody	Biolegend	Cat# 506327 RRID: AB_10900823
F4/80 Monoclonal Antibody (BM8), FITC	Invitrogen	Cat# 11-4801-82 RRID: AB_2637191
FITC anti-mouse CD14 Antibody	Biolegend	Cat# 123307 RRID: AB_940578
HRP Goat Anti-Mouse IgG H+L	Biotium	Cat# 20400-1mg RRID: AB_3083795
HRP Goat Anti-Rabbit IgG (H+L)	Biotium	Cat# 20402-1mg RRID: AB_3083796
HRP Goat Anti-Rat IgG	Sigma Aldrich	Cat# A9037-1ML RRID: AB_258429
Mouse monoclonal anti-β-ACTIN	Sigma Aldrich	Cat# A1978 RRID: AB_476692
Mouse monoclonal anti-CHOP	Cell Signaling Technology	Cat# 2895 RRID: AB_2089254
Mouse monoclonal anti-phospho-IκBα (Ser32/36)	Cell Signaling Technology	Cat# 9246 RRID: AB_2267145
Mouse monoclonal anti-NF-κB p65	Santa Cruz	Cat# sc-8008 RRID: AB_628017
Mouse monoclonal anti-ZBP1	Adipogen	Cat# AG-20B-0010 RRID: 2490191
Rabbit monoclonal anti-BIP	Cell Signaling Technology	Cat# 3177 RRID: AB_2119845
Rabbit polyclonal anti-GPX4	Abcam	Cat# ab41787 RRID: AB_941790
Rabbit polyclonal anti-IκBα	Santa Cruz	Cat# sc-371 RRID: AB_2235952
Rabbit monoclonal anti-MIF	Cell Signaling Technology	Cat# 87501S RRID: AB_2943242
Rabbit monoclonal anti-phospho-NF-κB p65 (Ser536)	Cell Signaling Technology	Cat# 3033 RRID: AB_331284
Rabbit monoclonal anti-phospho-MLKL (Ser345)	Cell Signaling Technology	Cat# 37333 RRID: AB_2799112
Rat monoclonal anti-MLKL	Millipore	Cat# MABC604 RRID: AB_2820284
**Chemicals, peptides, and recombinant proteins**		
2-Propanol	Roth	Cat# 9866.5
3,3′5,5′ tetramethylbenzidine (TMB)	Sigma Aldrich	Cat# 87748
30% H_2_O_2_	Sigma Aldrich	Cat# 95313
ABT737	Cayman Chemical	Cat# 11501-1
Acetic Acid (CH_3_COOH)	Roth	Cat# 3738.2
Acetone	Sigma Aldrich	Cat# 179124
Acetonitrile hypergrade for LC-MS	Merck Millipore	Cat# 1.00029.2500
Amersham ECL Prime Western Blotting Detection Reagent	Cytiva	Cat# RPN2235
Ammonium carbonate	Merck Millipore	Cat# 207861-500G
Ammonium hydroxide solution	Merck Millipore	Cat# 221228-1L-A
Ammonium persulfate	Sigma Aldrich	Cat# 431532
Arg10 HCL	Silantes	Cat# 201604102
Benzonase HC	Merck Millipore	Cat# 71206
Birinapant	Selleck Chem	Cat# S7015
Bovine Serum Albumin (BSA)	Sigma Aldrich	Cat# A7030
BODIPY™ 581/591 C11	Invitrogen	Cat# D3861
Brefeldin A	Biolegend	Cat# 420601
CAA (2-Chloracetamid)	Merck Millipore	Cat# 79-07-2
cOmplete^™^, EDTA-free Protease Inhibitor Cocktail	Sigma Aldrich	Cat# 4693132001
DMEM, high glucose, no glutamine, no phenol red	Thermo Fisher	Cat# 31053028
DMEM, high glucose, GlutaMAX™ supplement	Thermo Fisher	Cat# 61965059
Dimethyl sulfoxide (DMSO)	PAN Biotech	Cat# P60-36720100
Dithiothreitol (DTT)	VWR	Cat# 441496P
Doxycycline	VWR	Cat# J63805.06
DRAQ7	Biolegend	Cat# 424001
eBioscience™ Fixable Viability Dye eFluor™ 780	Invitrogen	Cat# 65-0865-14
EDTA (Versene), 1%, in PBS, without Ca2+ and Mg2+	Genexxon Bioscience	Cat# C4263.0100
Emricasan	Hölzel	Cat# HY-10396
Erastin	Biomol	Cat# 5449/10
Ethanol 99%, absolute	Roth	Cat# 9065.3
Ethylenediaminetetraacetic acid (EDTA)	VWR	Cat# 1084520250
Ferrostatin-1	Cayman Chemicals	Cat# 17729
Formic acid	Honeywell/Fluka	Cat# 607-001-00-0
ITS+1	Sigma Aldrich	Cat# I2521
LPS from E. coli, Serotype EH100 (Ra) (TLRGRADE™) (Ready-to-Use)	Enzo	Cat# ALX-581-010-L001
Lys8 HCL	Silantes	Cat# 211604102
Lysyl Endopeptidase (LysC)	WAKO	Cat# 129-02541
Methanol-LC-MS grade	VWR	Cat# 1.06035.2500
Murine His-TNF-alpha	Walczak lab	Kupka et al. [115]
ML210	Tocris	Cat# 6429/10
*N, N, N*′, *N*′-Tetramethylethylenediamine (TEMED)	Sigma	Cat# T9281
Necrostatin-1s	Abcam	Cat# ab221984
PBS, pH 7.4	Thermo Fisher	Cat# 10010056
Penicillium Streptomycin (P/S)	Sigma	Cat# P4333-100ML
PhosSTOP^™^	Sigma	Cat# 4906837001
Ponceau S	Sigma	Cat# P3504
Protease inhibitor	Sigma Aldrich	Cat# 4693132001
Recombinant Murine M-CSF	Peprotech	Cat# 315-02
RIPA buffer	Thermo Fisher	Cat# 89901
ROTIPHORESE^®^ Gel 30	Roth	Cat# 3029.2
RPMI 1640 medium	Thermo Fisher	Cat# 21875091
RSL3	Selleck Chem	Cat# S8155
S63845	Selleck Chem	Cat# S8383
SILAC DMEM FBS Kit	Silantes	Cat# 282006500
Skim milk powder	VWR	Cat# A0830
Sodium Acetate Trihydrate (CH3COONa.3H2O)	VWR	Cat# 1062671000
Sodium Azide	Sigma	Cat# S2002-100G
Sodium Chloride (NaCl)	VWR	Cat# 1064045000
Sodium dodecyl sulfate (SDS)	Sigma Aldrich	Cat# L3771-100G
STY-BODIPY	Cayman Chemical	Cat# Cay27089-500
Sulfuric acid 10% (H_2_SO_4_)	VWR	Cat# 95441000
SuperSignal^TM^ West Femto Maximum Sensitivity Substrate	Thermo Fisher	Cat# 34095
Tamoxifen (4OHT)	Sigma Aldrich	Cat# H7904-5MG
TEAB	Sigma Aldrich	Cat# T7408
Tris	VWR	Cat# 1083872500
Tris-HCl	VWR	Cat# 648313-250
Triton x100	VWR	Cat# 1086031000
Trypsin-EDTA (%0.25)	Gibco	Cat# 25200056
Tween 20	VWR	Cat# 0777-1L
Urea	Sigma Aldrich	Cat# U1250
Valine-d8	CK Isotopes	Cat# DLM-488
**Critical commercial assays**		
8-OHdG ELISA	BioVision	Cat# K4160-100
Bicinchoninic acid (BCA) protein assay	Biorad	Cat# 774985
Click Chemistry Capture Kit	Jena Bioscience	Cat# CLK-1065
CytoTox 96® Non-Radioactive Cytotoxicity Assay	Promega	Cat# G1780
LunaScript RT SuperMix Kit	NEB	Cat# E3010L
Luna Universal qPCR Master Mix	NEB	Cat# M3003E
Mouse CXCL1/KC DuoSet ELISA	R&D Systems	Cat# DY453
Mouse CXCL2/MIP-2 DuoSet ELISA	R&D Systems	Cat# DY452
Mouse IL-1beta/IL-1F2 DuoSet ELISA	R&D Systems	Cat# DY401
Mouse IL-6 DuoSet ELISA	R&D Systems	Cat# DY406
Mouse MIF DuoSet ELISA	R&D Systems	Cat# DY1978
Mouse TNF-alpha DuoSet ELISA	R&D Systems	Cat# DY410
NucleoSpin RNA Kit	Macherey-Nagel	Cat# 740955250
Prostaglandin E2 Parameter Assay Kit	R&D Systems	Cat# KGE004B
Proteome Profiler Mouse XL Cytokine Array	R&D Systems	Cat# ARY028
*RealTime-Glo™ Extracellular ATP Assay*	*Promega*	*Cat# GA5010*
**Deposited data**		
Proteomics Data	PRIDE database	PXD050979
Lipidomics	Metabolights database	MTBLS9798
Metabolomics	National Metabolomics Data Repository [116]	ST003635
Bulk RNA seq	NCBI Gene Expression Omnibus	GSE262664
**Experimental models: Cell lines**		
GPX4 Control mouse small cell lung cancer tumor line RP252.7	Bebber et al. [35]	N/A
GPX4 KO mouse small cell lung cancer tumor line RP252.7	Bebber et al. [35]	N/A
Tamoxifen-inducible GPX4 KO mouse embryonic fibroblast cell line Pfa1	Seiler et al. [31]	N/A
mFSP1 overexpressing, tamoxifen-inducible GPX4 KO mouse embryonic fibroblast cell line Pfa1	Doll et al. [9]	N/A
Immortalized murine bone marrow-derived macrophages (iBMDM line CL13)	De Nardo et al. [65]	N/A
ZBP1i MEFs	Jiao et al. [36]	N/A
Empty vector-inducible MEFs	Jiao et al. [36]	N/A
pBMDMs	von Karstedt lab	This paper
PMLFs	von Karstedt lab	This paper
**Experimental models: Organisms/strains**		
*Mus Musculus:* C57BL/6 N in-house strain was used for the generation of primary cells	CECAD in vivo research facility at the Cologne University Medical Center	Jackson Laboratory Cat# 005304 RRID: IMSR_JAX:005304
**Oligonucleotides**		
Please refer to Supplementary Table 6		
**Software and algorithms**		
MaxQuant versions 1.5.3.8 and 2.0.3	Tyanova et al. [100]	https://cox-labs.github.io/coxdocs/maxquant_instructions.html
Perseus 1.6.15	Tyanova et al. [101]	https://cox-labs.github.io/coxdocs/perseus_instructions.html
Uniprot reference genome Last access 26.08.2020	The UniProt consortium https://www.uniprot.org	ID: UP000000589 https://www.uniprot.org/proteomes/UP000000589
Tracefinder version 5.0	Thermo Fisher	https://www.thermofisher.com/order/catalog/product/OPTON-31001
Compound Discoverer version 3.2	Thermo Fisher	https://www.thermofisher.com/order/catalog/product/OPTON-31055
Flowjo version 10.6.2	BD Life Sciences	https://www.flowjo.com/solutions/flowjo/downloads/previous-versions
The FUSION Solo S software	Vilber	https://www.vilber.com
SeeMS version 3.0.23277.0	Chambers et al. [117]	https://proteowizard.sourceforge.io/download.html
Prism version 10	GraphPad	https://www.graphpad.com
ChemDraw version 21.0	Revvity Signals/Perkin Elmer	RRID:SCR_016768 https://revvitysignals.com/products/research/chemdraw
Skyline software 23.1	Chambers et al. [117]	https://proteowizard.sourceforge.io/download.html
IncuCyte 2021B	Sartorius	https://www.sartorius.com/en/products/live-cell-imaging-analysis/live-cell-analysis-software
AccuCor algorithm	Xiaoyang Su	https://github.com/lparsons/accucor

All reagents, their sources, and unique identifiers used for this study are listed as shown.

### Targeted analysis of oxylipins by LC-MS/MS

200,000 Pfa1 MEFs were seeded on 10 cm dishes in the presence of DMSO, 1 µM tamoxifen, and 1 µM Fer-1 in 8 ml phenol-red free medium. Supernatants were collected upon centrifugation for 5 min at 3000 rpm and 4 °C, and 1 mL was subsequently snap frozen in liquid nitrogen. Sample preparation for oxylipin analysis was performed as previously described [103] with modifications. Medium samples were previously centrifuged to remove cellular debris. Briefly, 500 μL of supernatant was added with 100 µL of internal standard mixture (Table 2). Samples were vortexed and applied into solid-phase extraction (SPE) Strata C18-E columns (50 mg; 8B-S001-DAM, Phenomenex). The SPE columns were washed with 1 mL of 5% methanol, and the oxylipins were eluted using 100% methanol. Samples were dried under N2 gas and dissolved in 50 µL of methanol for analysis. The oxylipin analysis was carried out by Q-Exactive Plus (Thermo) interfaced with an ultra-high-performance liquid chromatography (Vanquish, Thermo). The chromatographic and mass spectrometry conditions for analysis of oxylipins were carried out as described, with modifications. Samples were loaded into a UPLC BEH shield Reversed Phase C18 column (2.1 × 100 mm; 1.7 μm; Waters) with a flow rate of 0.4 mL/min and oven temperature maintained at 40 °C. The mobile phase A consisted of formic acid/water/acetonitrile (0.02:70:30), while mobile phase B composed of acetonitrile/isopropanol (70:30). Oxidized lipids were separated by a gradient as follows: from 0.1 to 40% B over the first 3.5 min, from 40 to 75% B from 3.5 to 6.0 min, from 75 to 99% B from 6.0 to 6.5 min, hold at 99% B from 6.5 to 10.5 min, decreased from 99 to 0.1% B during 10.5–11 min, and hold at 0.1% B from 11–15 min. The mass spectrometry was operated in negative ionization mode. The ESI parameters used in this analysis were: sheath gas (30 au), auxiliary gas (10 au), spray voltage (2.5 kV), ion transfer temperature: 320 °C, S-lens RF level: 55%, and aux gas heater temperature: 120 °C. Data for lipid molecular species identification and quantification were obtained by a parallel reaction monitoring (PRM) experiment. PRM data was acquired with a resolution setting of 17,500 at 200 m/z, AGC target 5e4 counts, Maximum IT 50 ms, isolation window 1.5 m/z. Collision energy (CE) was individually optimized for each ion (Table 3). Specific fragment ions monitored for each oxidized lipid species were manually identified using SeeMS and ChemDraw software. Quantification was performed by monitoring the peak area of specific fragments for each analyte using Skyline software. The area ratio obtained for each oxidized lipid was calculated by dividing the peak area of the lipid by the corresponding internal standard. The concentration of lipid species was calculated by applying the area ratio in a calibration curve constructed for each analyte. The concentration of oxylipins was expressed in pg/mL of medium.

**Table 2:** General information about internal standards used for oxylipin quantification.

Precursor ion [M-H]^-^	Specific fragment [M-H]^-^	Internal standard	RT (min)	CE (eV)	Analytes assigned
299.3	198.1579	13-HODE-d4	6.35	19	10
317.3	185.1516	12(13)-DiHOME-d4	5.36	26	2
355.2	193.1536	PGE_2_-d4	3.47	23	18
356.2	115.0401	LXA_4_-d5	3.88	20	1
364.2	153.0921	Protectin-D1-d5	4.81	20	3

*RT* retention time, *CE* collision energy.

**Table 3:** General information about oxylipins monitored.

Precursor ion [M-H]^-^	Specific fragment [M-H]^-^	Oxylipin	RT (min)	*R*^2^	LOD (pg)	LOQ (pg)	CE (eV)
295.2	171.1027	9-HODE	6.35	0.999	1.0	1.0	25
295.2	195.1391	13-HODE	6.29	1.000	1.0	1.0	25
313.2	183.1391	12(13)-DiHOME	5.27	1.000	0.5	0.5	23
313.2	201.1132	9(10)-DiHOME	5.43	1.000	0.5	0.5	23
315.2	271.2067	15-deoxy-Δ^12,14^-PGJ_2_	5.92	0.999	0.5	0.5	13
319.2	115.0401	5-HETE	6.81	0.999	0.5	3.9	19
319.2	167.1078	11-HETE	6.52	1.000	0.5	0.5	19
319.2	179.1078	12-HETE	6.60	1.000	1.0	1.0	19
319.2	113.0972	15-HETE	6.38	1.000	2.0	7.8	19
321.2	181.1234	12-HETrE	6.79	1.000	0.5	0.5	18
321.2	221.1547	15-HETrE	6.65	1.000	0.5	0.5	18
333.2	189.1285	PGA_2_	4.36	1.000	4.9	4.9	17
333.2	233.1183	PGJ_2_	4.41	0.998	2.4	4.9	17
333.2	235.1340	PGB_2_	4.44	0.999	4.9	4.9	17
333.2	271.2067	15-deoxy-Δ^12,14^-PGD_2_	5.12	0.999	4.9	4.9	17
343.2	234.1261	14-HDoHE	6.47	0.999	11.7	11.7	18
343.2	245.1547	17-HDoHE	6.37	0.999	11.7	11.7	18
349.2	235.0976	15-keto-PGE_2_	3.56	1.000	2.4	2.4	15
349.2	269.1911	PGE_3_	2.83	0.999	1.5	1.5	15
349.2	269.1911	PGD_3_	2.99	0.999	1.5	1.5	15
351.2	271.2067	PGE_2_	3.34	1.000	2.4	2.4	17
351.2	271.2067	PGD_2_	3.52	0.996	4.9	4.9	17
351.2	115.0401	LXA_4_	3.90	1.000	1.5	1.5	17
353.2	235.1340	PGD_1_	3.51	0.997	2.4	2.4	21
353.2	309.2071	PGF_2_α	3.19	0.999	4.9	4.9	21
353.2	115.0401	8,12-*iso*-iPF_2_α -VI	3.87	1.000	0.7	1.5	21
355.2	311.2228	13,14-dihydro-PGF_2_α	3.60	0.999	4.9	4.9	23
355.2	311.2228	PGF1α	3.19	0.996	4.9	4.9	23
359.2	153.0921	Protectin-D1	4.83	1.000	1.5	1.5	20
359.2	250.1211	Maresin-1	4.96	1.000	5.9	11.7	20
367.2	143.0714	6-keto-PGE_1_	2.20	1.000	4.9	4.9	21
369.2	245.1911	6-keto-PGF_1_α	1.94	1.000	4.9	9.8	26
369.2	267.1602	6,15-diketo-13,14-dihydro-PGF_1_α	2.76	1.000	11.7	46.9	26
375.2	215.1441	Resolvin-D1	3.85	1.000	1.5	11.7	20

*RT* retention time, *LOD* limit of detection, *LOQ* limit of quantification, *CE* collision energy.

### Intracellular and supernatant metabolomes of ferroptotic Pfa1 cells using HPLC-MS/MS

#### Metabolite Extraction

For metabolomics analysis of ferroptotic supernatants, Pfa1 MEFs were seeded on 6-well plates in the presence of DMSO, 1 µM tamoxifen, 1 µM Fer-1, or 1 µM Tamoxifen and 1 µM Fer-1 (25,000 cells per well; pentaplicates per condition), and samples were harvested at 24 h (cells + supernatant), 48 h (cells + supernatant), and 72 h (supernatant only). For sample harvest, supernatants were centrifuged at full speed and 4 °C for 5 min, and 50 µL of the cleared supernatants were added to 350 µL ice-cold extraction solution (50% methanol, 30% acetonitrile (both from Fisher Scientific)), 20% ultrapure water, 5 µM valine-d8 (CK isotopes) on dry ice. Cells were washed twice with ice-cold PBS and subsequently incubated with 100 µL extraction solution for 20 min in a dry ice/methanol bath, followed by scraping. Supernatants and cell samples were then stirred vigorously in a thermomixer at full speed and 4 °C followed by 20 min centrifugation at 4 °C and full speed. Cleared samples were stored at −80 °C and submitted for further metabolomic analysis.

Chromatographic separation of metabolites was achieved using a Millipore Sequant ZIC-pHILIC analytical column (5 µm, 2.1 × 150 mm) equipped with a 2.1 × 20 mm guard column (both 5 mm particle size) with a binary solvent system. Solvent A was 20 mM ammonium carbonate, 0.05% ammonium hydroxide; Solvent B was acetonitrile. The column oven and autosampler tray were held at 40 °C and 4 °C, respectively. The chromatographic gradient was run at a flow rate of 0.200 mL/min as follows: 0–2 min: 80% B; 2–17 min: linear gradient from 80% B to 20% B; 17–17.1 min: linear gradient from 20% B to 80% B; 17.1–23 min: hold at 80% B. Samples were randomized and the injection volume was 5 µl. A pooled quality control (QC) sample was generated from an equal mixture of all individual samples and analyzed interspersed at regular intervals.

#### Metabolite measurement by LC-MS

Metabolites were measured with Vanquish Horizon UHPLC coupled to an Orbitrap Exploris 240 mass spectrometer (both Thermo Fisher Scientific) via a heated electrospray ionization source. The spray voltages were set to +3.5 kV/−2.8 kV, RF lens value at 70, the heated capillary held at 320 °C, and the auxiliary gas heater held at 280 °C. The flow rate for sheath gas, aux gas, and sweep gas was set to 40, 15, and 0, respectively. For MS1 scans, mass range was set to m/z = 70–900, AGC target set to standard, and maximum injection time (IT) set to auto. Data acquisition for experimental samples used full scan mode with polarity switching at an Orbitrap resolution of 120,000. Data acquisition for untargeted metabolite identification was performed using the AcquireX Deep Scan workflow, an iterative data-dependent acquisition (DDA) strategy using multiple injections of the pooled sample. DDA full scan-ddMS2 method for AcquireX workflow used the following parameters: full scan resolution was set to 60,000, fragmentation resolution to 30,000, fragmentation intensity threshold to 5.0e3. Dynamic exclusion was enabled after 1 time, and the exclusion duration was 10 s. Mass tolerance was set to 5 ppm. The isolation window was set to 1.2 m/z. Normalized HCD collision energies were set to stepped mode with values at 30, 50, and 150. The fragmentation scan range was set to auto, AGC target at standard, and max IT at auto. Mild trapping was enabled. Metabolite identification was performed in the Compound Discoverer software (v 3.2, Thermo Fisher Scientific). Metabolite identities were confirmed using the following parameters: (1) precursor ion m/z was matched within 5 ppm of theoretical mass predicted by the chemical formula; (2) fragment ions were matched within 5 pm to an in-house spectral library of authentic compound standards analyzed with the same ddMS2 method with a best match score of over 70; (3) the retention time of metabolites was within 5% of the retention time of a purified standard run with the same chromatographic method.

#### Data analysis

Chromatogram review and peak area integration were performed using the Tracefinder software (v 5.0, Thermo Fisher Scientific), resulting in raw peak data. Next, we used the R package MetaProViz (v.2.0.1) for all subsequent data analysis. First, using the MetaProViz Preprocessing() function, the raw peak area for each detected metabolite was subjected to the “Modified Filtering Rule” [104], half minimum missing value imputation, and normalized against the total ion count (TIC) of that sample to correct any variations introduced from sample handling through instrument analysis. In case of media supernatant samples MetaProViz Preprocessing() function parameter “CoRe” was set to “TRUE”, and the pre-processed mean value of each metabolite detected in the fresh culture medium (incubated in the absence of cells) was subtracted from the metabolites detected in the supernatant samples. Testing for outliers based on Hotelling’s T2 test [105], with a 0.99 confidence interval. Afterwards, differential metabolomics analysis was performed using the MetaProViz DMA() function to calculate the Log2FC in case of intracellular samples or the Log2(Distance) in case of supernatant media samples (parameter CoRe = TRUE). The *p*-value and *t*-test were calculated using t.test and adjusted using fdr. The results were visualized using MetaProViz VizVolcano and VizHeatmap, relying on the dependencies EnhancedVolcano (v. 1.20.0) [106] and pheatmap (v. 1.0.12) [107].

### Isolation of RNA and quantitative RT-PCR

Total RNA from tissues was isolated using the NucleoSpin RNA kit according to the manufacturer’s protocol. RNA concentration was determined using the Nanodrop 8000 spectrophotometer. cDNA synthesis from the isolated RNA was reverse transcribed using the LunaScript RT SuperMix Kit following the protocol provided by the manufacturer. Real-time qPCR was performed in quadruplets on the Quant Studio 5 qRT-PCR machine. Relative expression of gene transcripts was analyzed via the 2-ΔCT or the 2-ΔΔCT method to the reference gene Glycerinaldehyde-3-phosphate Dehydrogenase (GAPDH). Primer sequences can be found in Table S6.

### RNA sequencing

For RNA sequencing, 500,000 primary bone marrow-derived macrophages (pBMDMs) were plated in 6-well plates with M-CSF [25 ng/ml] at day 6 post-differentiation. Fresh, filtered supernatants with or without 4OHT [1 μM], Ferrostatin-1 (Fer-1) [1 μM], were incubated with pBMDMs for 24 h. The next day, cells were washed with PBS, and RNA was isolated using the NucleoSpin RNA kit (740955.5, Macherey-Nagel) according to the manufacturer’s instructions. cDNA libraries amplified from the 3′ UTR were generated from total RNA using the Lexogen QuantSeq kit (Lexogen, Austria) according to the standard protocol and sequenced with a 50-bp single-end protocol on an Illumina HiSeq4000 sequencer (Illumina, USA).

Primary data analysis was conducted using the RNA-seq pipeline from the nf-core suite (v3.7) [108]; sequencing reads were aligned to the GRCh38 (v103) human reference genome using STAR (v2.7.10a) [109]. Gene quantification was conducted using Salmon (v1.5.2) [110]. The pipeline was executed with default parameters. Downstream differential expression analysis was performed using DESeq2 (v1.36.0) [111], with default parameters. To enhance the accuracy of fold-change estimation, we included mouse ID as a batch effect in the design matrix. For some comparisons (Fer-1 vs. Ctrl and 4OHT vs. Ctrl), the original *p*-values inferred by DESeq2 revealed significant deviation from the expected uniform null distribution, suggesting low sensitivity. To correct for this, we recomputed the raw *p*-values using fdrtool (v1.2.17) [112] to increase the power of the differential expression procedure while maintaining efficient control for false discovery. Subsequently, the Benjamini-Hochberg procedure was applied to correct the *p*-values for multiple tests.

GO enrichment analysis was conducted using gprofiler2 (v0.2.2) [113]. The selection criteria focused on differentially expressed genes, as defined above. Using ordered gene query and gProfiler’s “g_SCS” method for *p*-value adjustment, which accounts for the hierarchical structure of GO terms, enriched GO terms were identified among the differentially expressed genes.

We defined genes as differentially expressed if they exhibited an absolute log2FoldChange greater than 1 and an adjusted *p*-value lower than 0.05. For the heatmap generation, we focused exclusively on genes identified as differentially expressed (DEGs) in the 4OHT versus Ctrl comparison. These DEGs were ranked by the absolute log2 of fold change, and algorithmically predicted genes were excluded, leaving only those validated through experimental methods. Given that we have only three replicates per condition, to minimize the risk of false positives, we retained only genes that demonstrated non-zero counts per million (CPM) in all three replicates of at least one experimental condition. Subsequently, we selected the top 25 upregulated and the top 25 downregulated genes for the heatmap visualization. Additionally, we applied the variance stabilization transformation procedure from DESeq2 for normalization of the counts, setting the ‘blind’ parameter to FALSE, to account for design peculiarities as recommended by the DESeq2 authors. To mitigate the mouse batch effect, we utilized the function removeBatchEffect from the R package limma [114].

### Flow cytometry

pBMDMs: Fresh or frozen bone marrow cells were used to generate pBMDMs as previously described [97] using M-CSF [25 ng/ml] in the medium. At day 6 post-differentiation, 500,000 pBMDMs were seeded in 6-well plates and incubated with non-ferroptotic or ferroptotic supernatants for 24 h to confirm the differentiation. Macrophages were removed from the plates with ice-cold PBS and stained with the following antibodies/dyes: fixable viability dye eFluor™ 780, APC anti-mouse/human CD11b, FITC F4/80 Monoclonal Antibody (BM8). For intracellular TNFα staining, at day 6, 500,000 pBMDMs were seeded in 6-well plates and incubated with non-ferroptotic or ferroptotic supernatants containing IFN gamma [25 ng/ml], LPS [10 ng/ml], and Brefeldin A [5 μg/ml] for 6 h. Macrophages were removed from the plates with ice-cold PBS and stained with the following antibodies/dyes: fixable viability dye eFluor™ 780, APC anti-mouse/human CD11b, FITC F4/80 Monoclonal Antibody (BM8), and Brilliant Violet 421™ anti-mouse TNF-α.

Pfa1: 5000 Pfa1 MEFs were plated in 24-well plates and treated with or without 4OHT [1 μM], Ferrostatin-1 (Fer-1) [1 μM], and BODIPY C11 [5 μM]. 30 min before lysing at different time points, BODIPY C11 was added to each well. Cells were washed, detached, and the cell pellet was then resuspended in 200 μl of PBS with 2% FCS and [1 μg/ml] propidium iodide (PI).

Flow cytometry data were acquired on the BD LSRFortessa (BD Biosciences) and analyzed with Flowjo V10.6.2.

### Live cell imaging (IncuCyte)

1000 Pfa1, Pfa1-mFSP1-OE MEFs were plated in 96-well plates and treated with or without 4OHT [1 μM], and Ferrostatin-1 (Fer-1) [1 μM]. 5000 lung fibroblasts were plated in 96-well plates and treated with or without RSL3 [1 μM], erastin [10 μM], ferrostatin-1 (Fer-1) [1 μM], TNFα [20 ng/ml], birinapant [1 μM], emricasan [2,5 μM], nec1s [10 μM]. 5000 ZBP1i or empty vector-inducible MEFs were plated in 96-well plates and pre-treated with or without doxycycline [1 μg/ml] and treated with or without emricasan [2,5 μM]. For dead cell quantification, DRAQ7 [100 mM] was used. For lipid ROS quantification, STY-BODIPY [1 μM] was used. Cells were imaged every 2 h using the 10× objective within the IncuCyte SX5 live cell imaging system (Sartorius). Analysis for confluence, DRAQ7-positive (dead), reduced- and oxidized-BODIPY-positive cells was performed using the Software IncuCyte 2021B (Sartorius).

### Western blotting

Cells were washed once with PBS and lysed in RIPA buffer containing protease and phosphatase inhibitors on ice or overnight at −20 °C. Protein concentration was determined using bicinchoninic acid (BCA) protein assay according to the manufacturer’s instructions. Equal amounts of protein were mixed with a final concentration of 1× reducing sample buffer and 200 mM DTT. Cell lysates were heated for 10 min at 80 °C in order to speed up the process of denaturation. Gel electrophoresis of proteins was performed using the Mini-PROTEAN® Tetra Cell System by Biorad. Trans-Blot® Turbo Transfer System (Biorad) was used for the transfer of the proteins from the SDS gel to the nitrocellulose membrane. Membranes were blocked in PBS with 0.1% Tween 20 (PBS-T) with 5% (w/v) bovine serum albumin (BSA) for 1 h. Next, membranes were incubated with the primary antibodies overnight at 4 °C. The following day, membranes were washed and incubated with secondary antibody conjugated with horse radish peroxidase (HRP) at room temperature for 1 h. After the washing step, membranes were developed using Amersham ECL Prime Western Blotting Detection Reagent or SuperSignalTM West Femto Maximum Sensitivity Substrate. The FUSION Solo S system and software were used to image the membranes.

### LDH release assay

Pfa1 and mFSP1-overexpressing Pfa1 MEFs were seeded on a 24-well plate at 5000 cells per well and treated with or without 4OHT [1 μM] and Fer-1 [1 μM] as described above for 72 h. Zbp1i and vector-inducible MEFs were seeded on a 24-well plate at 35,000 cells per well and pre-treated with or without doxycycline 16 h [1 μg/ml] and treated with or without emricasan [5 μM] as described above for 24 h. Supernatants were collected at the respective indicated times post-treatment with different drug concentrations, and the presence of LDH was quantified by using CytoTox 96® Non-Radioactive Cytotoxicity Assay according to the manufacturer’s instructions.

### ELISA

Fibroblasts: Pfa1 and mFSP1-overexpressing Pfa1 MEFs were seeded on a 24-well plate at 5000 cells per well and treated with or without 4OHT [1 μM] and Fer-1 [1 μM] as described above for 72 h. Zbp1i and vector-inducible MEFs were seeded on a 24-well plate at 35,000 cells per well and pre-treated with or without doxycycline [1 μg/ml] 16 h and treated with or without emricasan [2.5 μM] as described above for 24 h. PMLFs were seeded on a 24-well plate at 35,000 cells per well and pre-treated with RSL3 [1 μM], erastin [10 μM], ferrostatin-1 [Fer-1, 1 μM], TNFα [20 ng/ml], birinapant [1 μM], emricasan [2.5 μM], and nec1s [10 μM].

pBMDMs/iBMDMs: pBMDMs or iBMDMs were seeded on the 6-well plate at 500,000 cells per well and incubated with ctrl. or GPX4 KO supernatants for 6 h with IFN gamma [25 ng/ml], after which LPS [10 ng/ml] was added for a total of 24 h.

Supernatants were collected and subsequently stored at −20 °C. Collected supernatants were used in 8-OHdG, CXCL1/KC, CXCL2/MIP-2, MIF, IL-6, TNFα, and IL-1β ELISAs according to the manufacturer’s instructions.

### Quantification and statistical analysis

Raw data from bulk RNA sequencing, proteomics, metabolomics, and lipidomics were processed as described above, and all other raw data were processed using Excel and GraphPad Prism. During the data acquisition for RNA sequencing, proteomics, metabolomics, and lipidomics, investigators were blinded to treatment condition (duration, compounds). For statistical testing and generation of figures, GraphPad Prism was used. For statistical testing, all experiments were repeated with a minimum of three biological replicates. Two groups were statistically compared using an unpaired *t*-test, and more than two groups were compared using one- or two-way ANOVA, where Tukey was used as a post hoc test. Unless indicated otherwise in the figure legend, results are presented as mean ± standard error of mean (SEM), and statistical significance is defined as depicted below.

ns: not significant; ∗*p* < 0.05; ∗∗, *p* < 0.01; ∗∗∗, *p* < 0.001; ∗∗∗∗, *p* < 0.0001.

## Supplementary information

Supplementary material

Original data file Western Blots

Table S1

Table S2

Table S3

Table S4

Table S5

Table S6

## Data Availability

All datasets are attached as supplemental tables (Tables S1–S6) and uploaded to corresponding public databases (see “reagents and tools” table for identifiers and databases). Further information and requests for resources and reagents should be directed to and will be fulfilled by the corresponding author, Silvia von Karstedt, e-mail: s.vonkarstedt@uni-koeln.de.
