# Supplementary material for: An atlas of ferroptosis-induced secretomes
Source: Cell Death Differ. 2025 Apr 25;32(11):1986–2008. doi: 10.1038/s41418-025-01517-4 (PMC12572367; doi:10.1038/s41418-025-01517-4)
Supplement: Supplementary file 1 — Supplementary material [file 41418_2025_1517_MOESM1_ESM.pdf]

Supplemental Information

An Atlas of Ferroptosis-induced Secretomes

F. Isil Yapici[1,2], Eric Seidel[1, 2], Alina Dahlhaus[1, 2], Josephine Weber[1,2], Christina Schmidt[3, 4, 5], Adriano de Britto Chaves Filho[6], Ming Yang[3, 4], Maria Nenchova[1,2], Emre Güngör[1, 2], Jenny Stroh[1,2], Ioanna Kotouza[1,2], Julia Beck[1,2], Ali T. Abdallah[2,7], Jan-Wilm Lackmann[4], Christina M. Bebber[1,2], Ariadne Androulidaki[1,2], Peter Kreuzaler[3], Almut Schulze[6], Christian Frezza[3, 4] & Silvia von Karstedt[1,2,8,#]

[1]University of Cologne, Faculty of Medicine and University Hospital Cologne, Department of Translational Genomics, Cologne, Germany.

[2]University of Cologne, Faculty of Medicine and University Hospital Cologne, CECAD Cluster of Excellence, University of Cologne, Cologne, Germany.

[3]University of Cologne, Faculty of Medicine and University Hospital Cologne, Institute for Metabolomics in Ageing, Cluster of Excellence Cellular Stress Responses in Aging-associated Diseases (CECAD), Cologne, Germany

[4]University of Cologne, Faculty of Mathematics and Natural Sciences, Institute of Genetics, Cluster of Excellence Cellular Stress Responses in Aging-associated Diseases (CECAD), Cologne, Germany

[5]Heidelberg University, Faculty of Medicine, and Heidelberg University Hospital, Institute for Computational Biomedicine, Heidelberg, Germany

[6]Division of Tumor Metabolism and Microenvironment, German Cancer Research Center (DKFZ) and DKFZ-ZMBH Alliance, Heidelberg, Germany

[7]University of Cologne, Faculty of Medicine, Institute of Medical Statistics and Computational Biology, Cologne.

[8]University of Cologne, Faculty of Medicine and University Hospital Cologne, Center for Molecular Medicine Cologne, Cologne, Germany.

[#]corresponding author

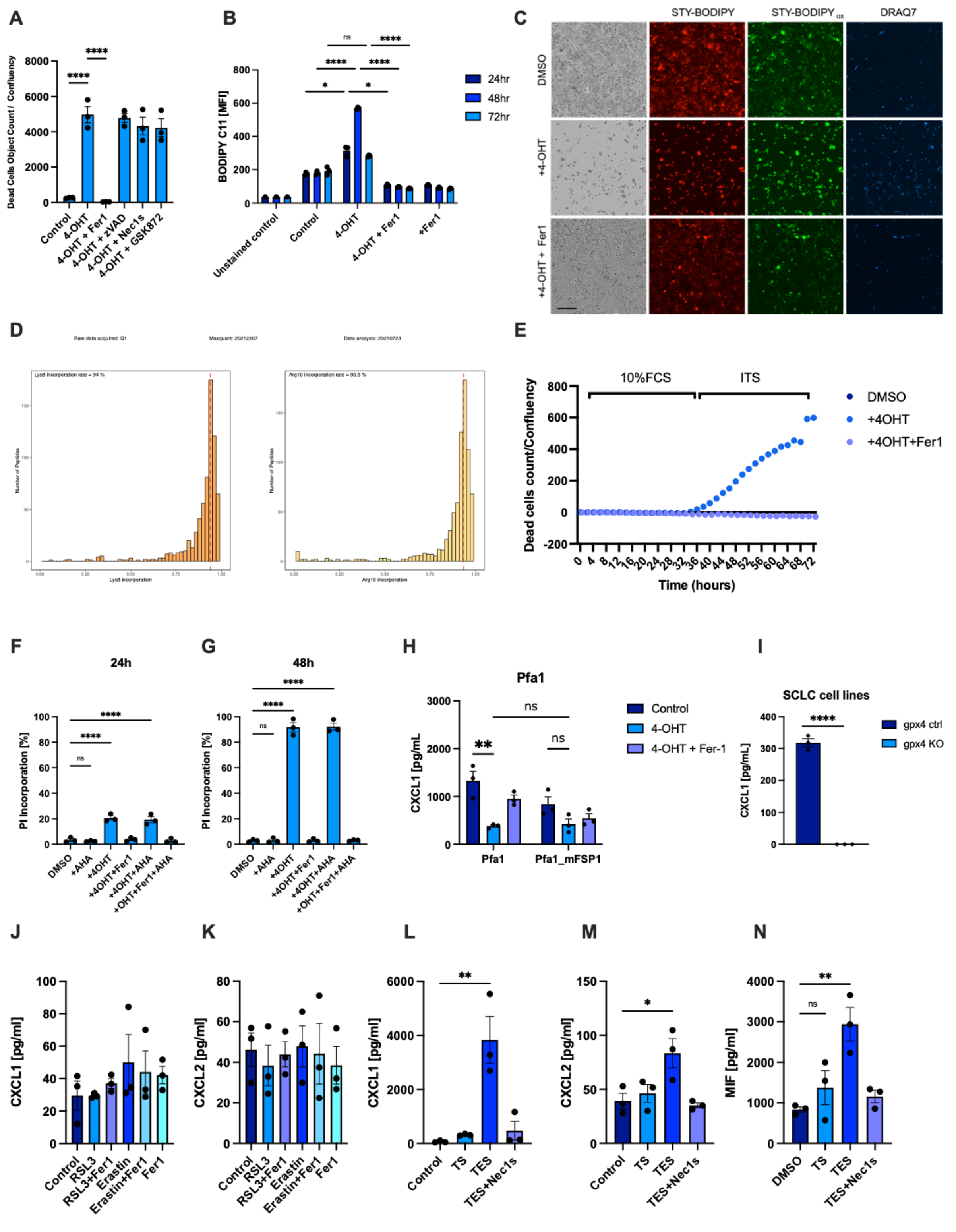

Figure S1. Necroptotic but not ferroptotic cells release CXCL1/2.

(A) Parental Pfa1 MEFs were treated +/- 4OHT [1µM] +/ - Ferrostatin-1 (Fer1) [1µM] +/ - zVAD [20µM] +/ - Nec1s [10µM] +/ - GSK872 [3,33µM] for 72 h. Dead cells were visualized using DRAQ7 [100nM] uptake. Images were acquired every 2 h using the IncuCyte SX5 bioimaging platform. Dead cell count was normalized to confluency.

(B) Cells as in A were treated +/- 4OHT [1µM] +/ - Fer1 [1µM] and were and stained for lipid ROS accumulation using BODIPY C11. Cells were analysed by flow cytometry. MFI, mean fluorescence intensity.

(C) Cells as in A were treated as in (B) and stained for lipid ROS accumulation using STY-BODIPY [1µM] for 72 h. Dead cells were visualized using DRAQ7 [100nM] Images were acquired every 2 h using the IncuCyte SX5 bioimaging platform. Levels of lipid peroxidation can be monitored by quantifying levels of co-oxidized STY-BODIPY (green, λex = 488 nm, λem = 495−540 nm) emitting green signals over reduced STY-Bodipy (red, λex = 561 nm, λem = 568−630 nm) emitting in the orange spectrum. Representative images are shown.

(D) Mass spectrometry integration test of Pfa1 MEFs cultured in SILAC medium containing Lys8 and Arg10 for 6 passages.

(E) Cells as in A were treated +/- 4OHT [1µM] +/ - Fer1 [1µM] in culturing medium containing 10% FCS for 40 h then changed to culturing medium containing only ITS+1 media supplement. DRAQ7 [100nM] was added to all wells to visualize dead cells. Cell death was normalized to confluency. Images were acquired every 2 h using the IncuCyte SX5 bioimaging platform.

(F) Cells as in A were treated +/- 4OHT [1µM] +/ - Fer1 [1µM] +/ - Azidohomoalanine (AHA) [100µM] for 24 h, cell death was measured with propidium iodide (PI) incorporation (%). Cells were analysed by flow cytometry.

(G) Cells as in A were treated as in (F) for 48 h, cell death was measured with propidium iodide (PI) incorporation (%). Cells were analysed by flow cytometry.

(H) Cells as in A or Pfa1 cells with stable FSP1 overexpression (Doll *et al*, 2019) (mFSP1) were treated with 4OHT [1µM] +/- Ferrostatin-1 [1µM] for 72 h. CXCL1 concentration within supernatants was quantified using ELISA.

(I) GPX4 control or GPX4-deficient SCLC cell lines (Bebber *et al*, 2021) were kept in the presence of Ferrostatin-1 [1µM]. Supernatants were collected 16 h after Ferrostatin-1 withdrawal. CXCL1 concentration within supernatants was quantified using ELISA.

(J) Primary mouse lung fibroblasts (PMLFs) were treated +/- RSL3 [1µM] +/- Fer1 [1µM] +/- Erastin [1µM] for 24 h. CXCL1 concentration within supernatants was quantified using ELISA.

(K) PMLFs were treated as in (H). CXCL2 concentration within supernatants was quantified using ELISA.

(L) PMLFs were treated +/- TNFα [20ng/ml] +/- Birinapant [1µM] (TS) +/- Emricasan [2,5µM] (TES) for 24 h. CXCL1 concentration within supernatants was quantified using ELISA.

(M) PMLFs were treated as in (J). CXCL2 concentration within supernatants was quantified using ELISA.

(N) PMLFs were treated as in (J). MIF concentration within supernatants was quantified using ELISA. Data information: (A, B, F-L) Graphs show data with means +/- SEM of at least three independent experiments. One- or two-way ANOVA was used to calculate p-values. ns: not significant; *: p<0.05; **: p<0.01; ***: p<0.001; ****: p<0.0001.

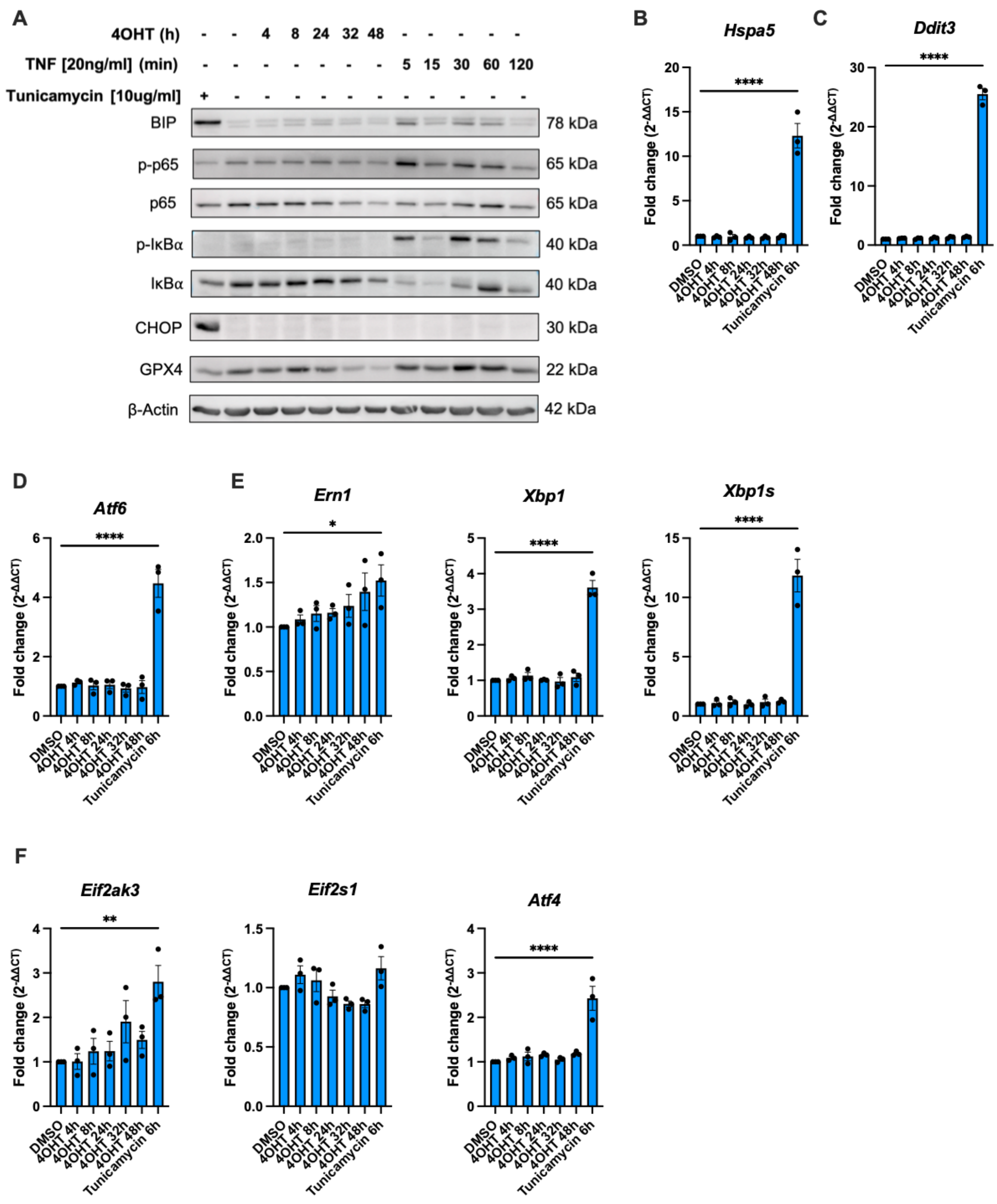

Figure S2. GPX4 deletion does not induce markers of ER stress.

(A) Pfa1 MEFs treated +/- 4OHT [1µM] for 4 h, 8 h, 24 h, 32 h and 48 h; +/- TNF alpha [20ng/ml] for 5, 15, 30, 60 and 120 min; +/ - Tunicamycin [20µM] for 6 h were subjected to Western blot analysis of the indicated proteins.

(B-F) Pfa1 MEFs treated +/- 4OHT [1µM] for 4 h, 8 h, 24 h, 32 h and 48 h; +/ - Tunicamycin [20µM] for 6 h. qPCR of the indicated transcripts was performed. (B-F) Graphs show data of means +/- SEM of at least three independent experiments. One- or two-way ANOVA was used to calculate p-values. ns: not significant; *: p<0.05; **: p<0.01; ***: p<0.001; ****: p<0.0001.

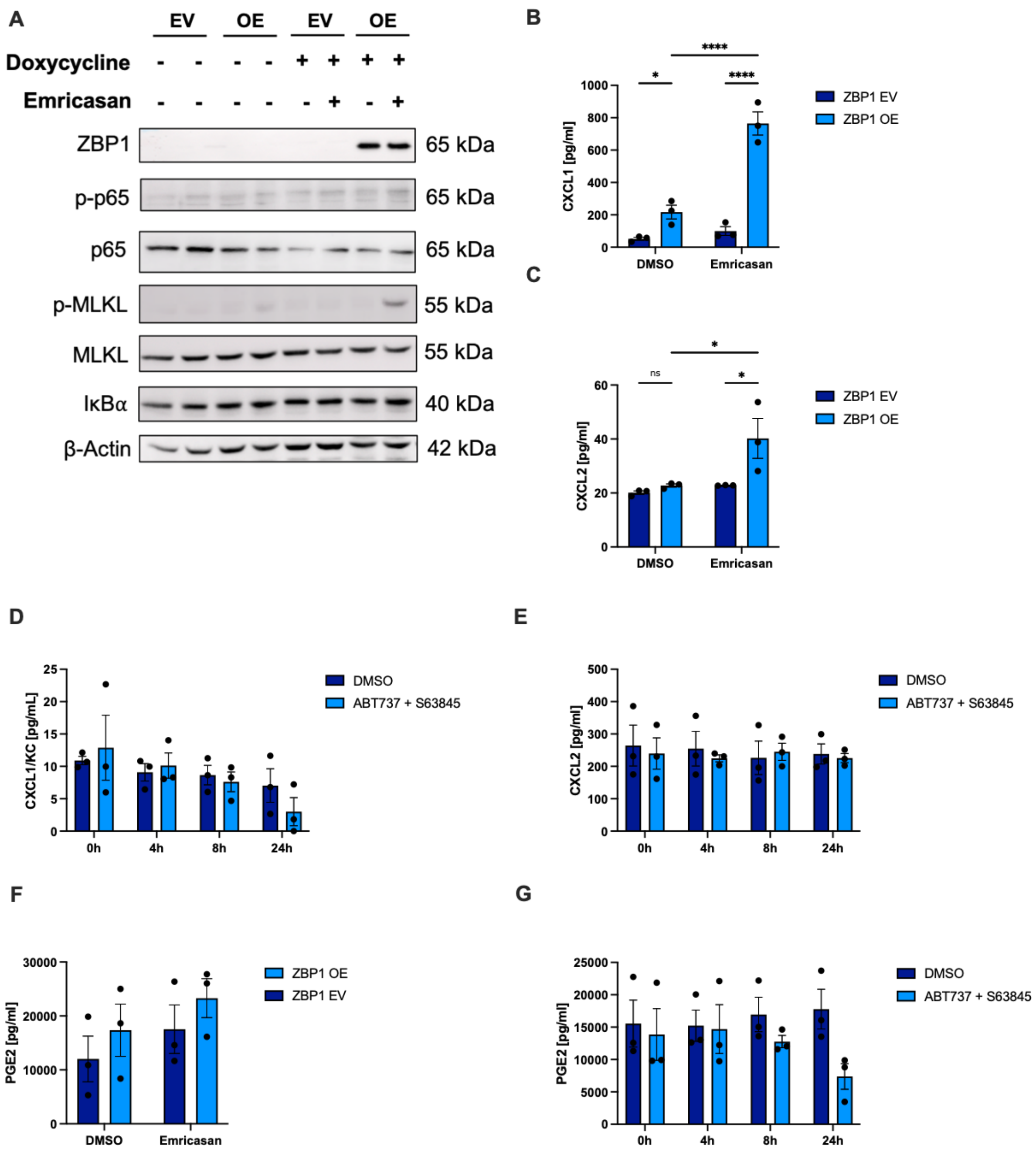

Figure S3. ZBP1-induced necroptosis induces the release of cytokines but lacks NF-κB activation.

(A) Empty vector (ZBP1 EV) ZBP1-inducible (ZBP1i) MEFs were pre-treated +/- doxycycline [1μg/ml] for 16 h and +/- Emricasan [2.5μM] for 4 h and were subjected to Western blot analysis of the indicated proteins.

(B) ZBP1-EV and ZBP1i MEFs were pre-treated +/- doxycycline [1μg/ml] for 16 h and +/- Emricasan [2.5μM] for 24 h. CXCL1 concentration within supernatants was quantified using ELISA.

(C) ZBP1-EV and ZBP1i MEFs cells were treated as in (B). CXCL2 concentration within supernatants was quantified using ELISA.

(D) ZBP1-EV and ZBP1i MEFs were treated +/- ABT737 [1μM] and S63845 [1μM] for 4 h, 8 h, and 24 h. CXCL1 concentration within supernatants was quantified using ELISA.

(E) ZBP1-EV and ZBP1i MEFs were treated as in (D). CXCL2 concentration within supernatants was quantified using ELISA.

(F) ZBP1-EV and ZBP1i MEFs were treated as in (D). PGE2 concentration within supernatants was quantified using ELISA.

(G) ZBP1-EV and ZBP1i MEFs were treated as in (B). PGE2 concentration within supernatants was quantified using ELISA. (B-E) Graphs show data of means +/- SEM of at least three independent experiments. One- or two-way ANOVA was used to calculate p-values. ns: not significant; *: $p<0.05$; **: $p<0.01$; ***: $p<0.001$; ****: $p<0.0001$.

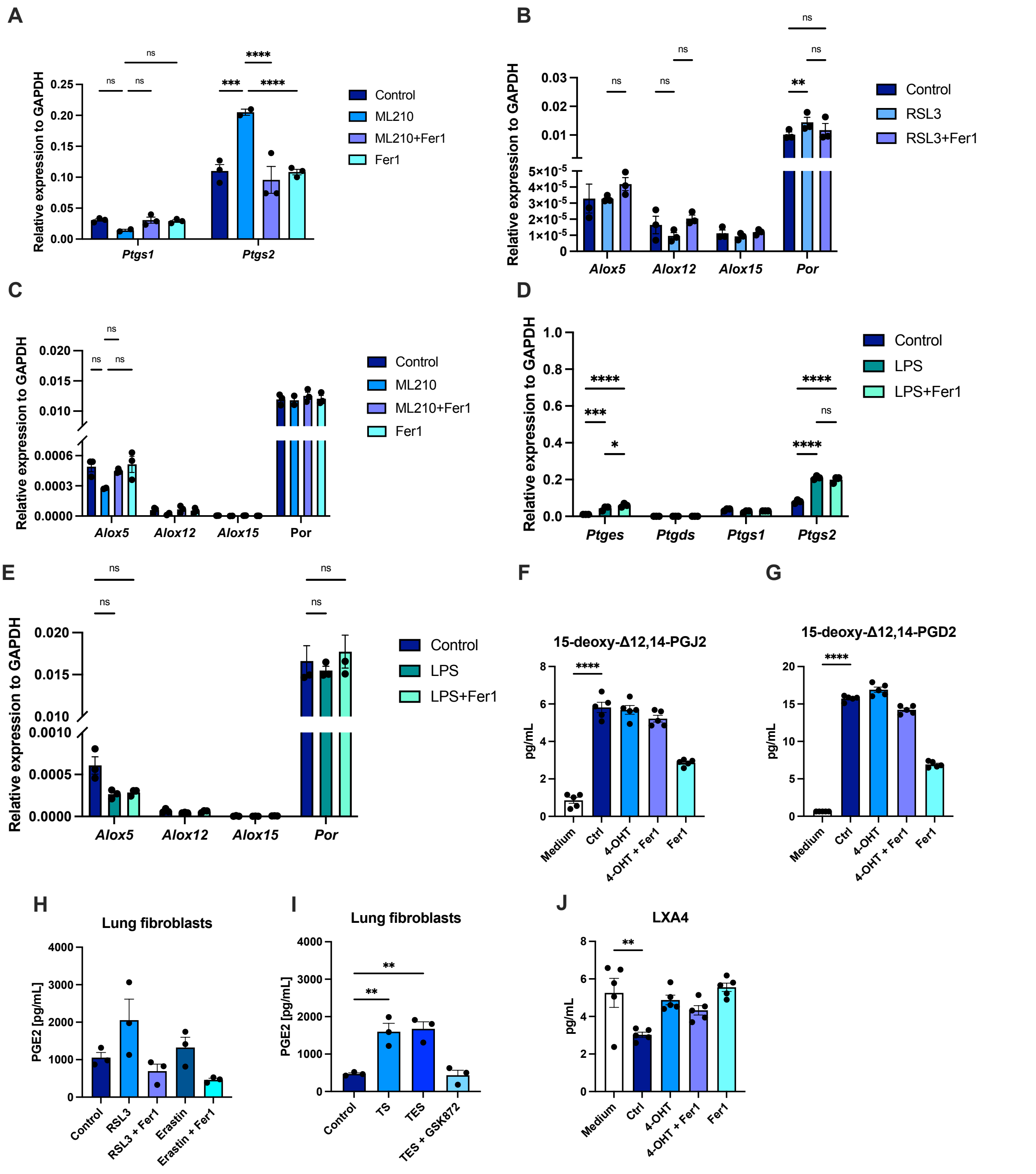

Figure S4. LPS induces *Ptgs2* independently of lipid ROS.

(A) Primary mouse lung fibroblasts (PMLFs) were treated +/- ML210 [0.5µM] +/- Fer1 [1µM] for 8 h. qPCR of the indicated transcripts was performed.

(B) Primary mouse lung fibroblasts (PMLFs) were treated +/- RSL3 [0.1µM] +/- Fer1 [1µM] for 6 h. qPCR of the indicated transcripts was performed.

(C) PMLFs were treated as in (A), qPCR of the indicated transcripts was performed.

(D) PMLFs were treated +/- LPS [10ng/ml] +/- Fer1 [1µM] for 6 h. qPCR of the indicated transcripts was performed.

(E) PMLFs were treated as in (D), qPCR of the indicated transcripts was performed.

(F-G) Parental Pfa1 MEFs were treated +/- 4OHT [1µM] +/- Ferrostatin-1 [1µM] for 72 h, supernatants (n=5 per condition) were collected, and concentrations of the indicated PGD2-derived oxylipins were quantified by mass spectrometry using standards as compared to media.

(H) PMLFs were treated with +/- RSL3 [1µM] +/- Fer1 [1µM] +/- Erastin [1µM] for 24 h. PGE_2 was quantified using ELISA.

(I) PMLFs were treated +/- TNFα [20ng/ml] +/- Birinapant [1µM] (TS) +/- Emricasan [2,5µM] +/- GSK872 [3,33µM] (TES) for 24 h. PGE_2 was quantified using ELISA.

(J) Parental Pfa1 MEFs were treated as in (F-G), concentration of lipoxin A4 was quantified by mass spectrometry using standards as compared to media. Data information: (A-E) Graphs show data with means +/- SEM of at least three independent experiments and performed using 5 biological replicates for lipidomic measurements. One- or two-way ANOVA was used to calculate p-values. ns: not significant; *: p<0.05; **: p<0.01; ***: p<0.001; ****: p<0.0001.

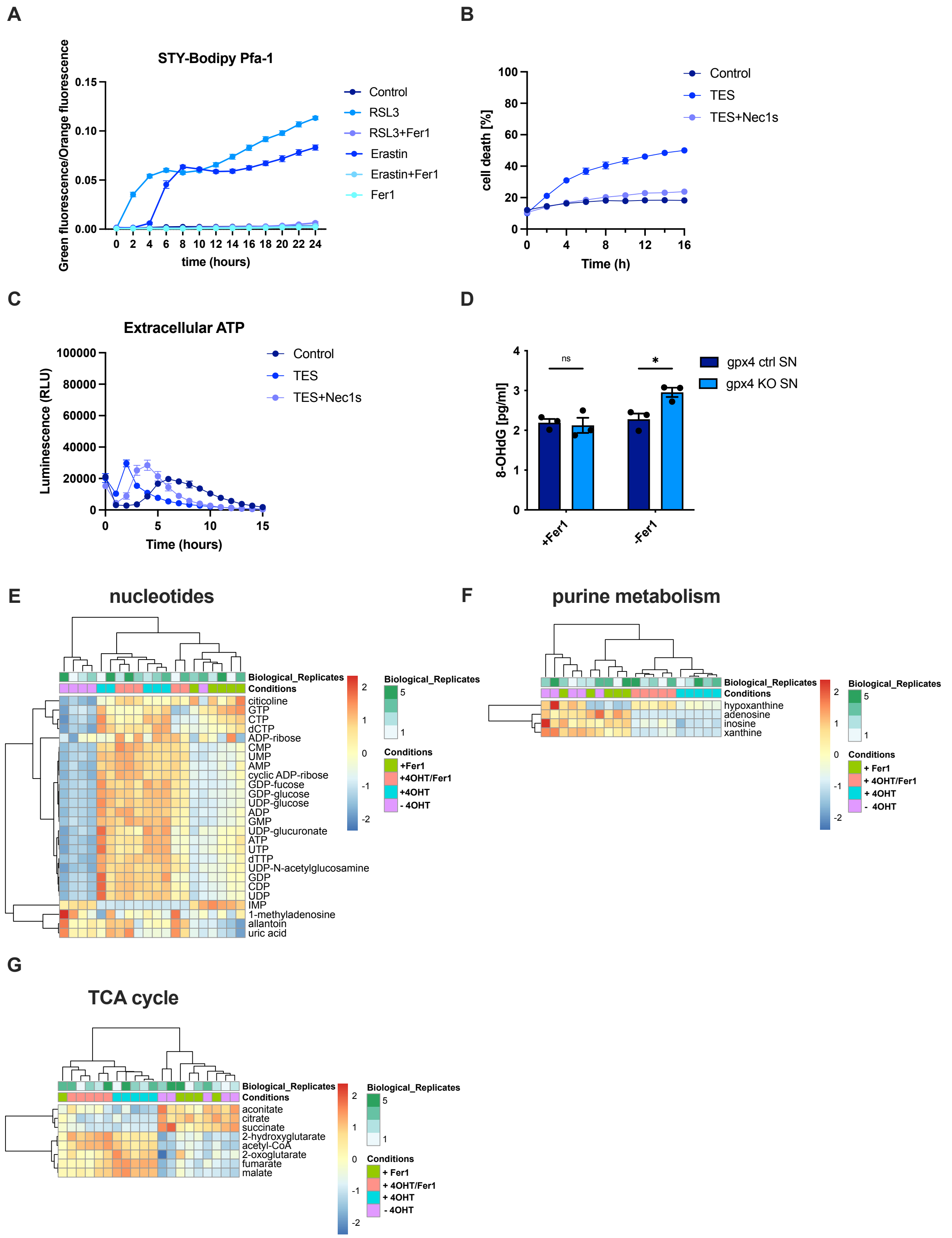

Figure S5. Necroptotic cells release limited amounts of ATP.

(A) Parental Pfa1 MEFs were treated with RSL3 [1µM] or Erastin [1µM] +/- Ferrostatin-1 [1µM] and stained for lipid ROS accumulation using STY-BODIPY [1µM] for 24 h. Levels of lipid peroxidation can be monitored by quantifying levels of co-oxidized STY-BODIPY (green, λex = 488 nm, λem = 495−540

nm) emitting green signals over reduced STY-Bodipy (red, λex = 561 nm, λem = 568−630 nm) emitting in the orange spectrum.

(B) PMLFs were treated +/- TNFα [20ng/ml] +/- Birinapant [1μM] +/- Emricasan [2,5μM] (TES) for 15 h. DRAQ7 [100nM] was added to all wells to visualize dead cells. % Cell death was normalized to confluency. Images were acquired every 2 h using the IncuCyte SX5 bioimaging platform.

(C) PMLFs were treated +/- TNFα [20ng/ml] +/- Birinapant [1μM] (TS) +/- Emricasan [2,5μM] (TES) for 15 h. ATP release was measured using the RealTime-Glo™ Extracellular ATP Assay and a luminescence plate reader. Relative Luciferase Units; RLU.

(D) GPX4 control (GPX4 ctrl SN) or GPX4 KO (GPX4 KO SN) SCLC cell lines (Bebber *et al*, 2021) were kept in the presence of Ferrostatin-1 [1μM]. Supernatants were collected 16 h after Ferrostatin-1 withdrawal and 8-OHdG was quantified using ELISA.

(E) Heatmap of nucleotides in cell pellets 30 h after 4OHT [1μM] stimulation of Pfa1 cells +/- Ferrostatin-1 [1μM]. Log2 fold change is shown.

(F) Heatmap of purine metabolism metabolites in cell pellets 30 h after 4OHT [1μM] stimulation of Pfa1 cells +/- Ferrostatin-1 [1μM].

(G) Heatmap of TCA cycle metabolites in cell pellets 30 h after 4OHT [1μM] stimulation of Pfa1 cells +/- Ferrostatin-1 [1μM]. Heatmap of significantly different (p=0.05) metabolites in media of Pfa1 cells +/- 4OHT [1μM] +/ - Ferrostatin-1 (Fer1) [1μM] for 48 h. Data information: (A-D) Graphs show data with means +/- SEM of at least three independent experiments and performed using 5 biological replicates for metabolomic measurements. One- or two-way ANOVA was used to calculate p-values. ns: not significant; *: p<0.05; **: p<0.01; ***: p<0.001; ****: p<0.0001.

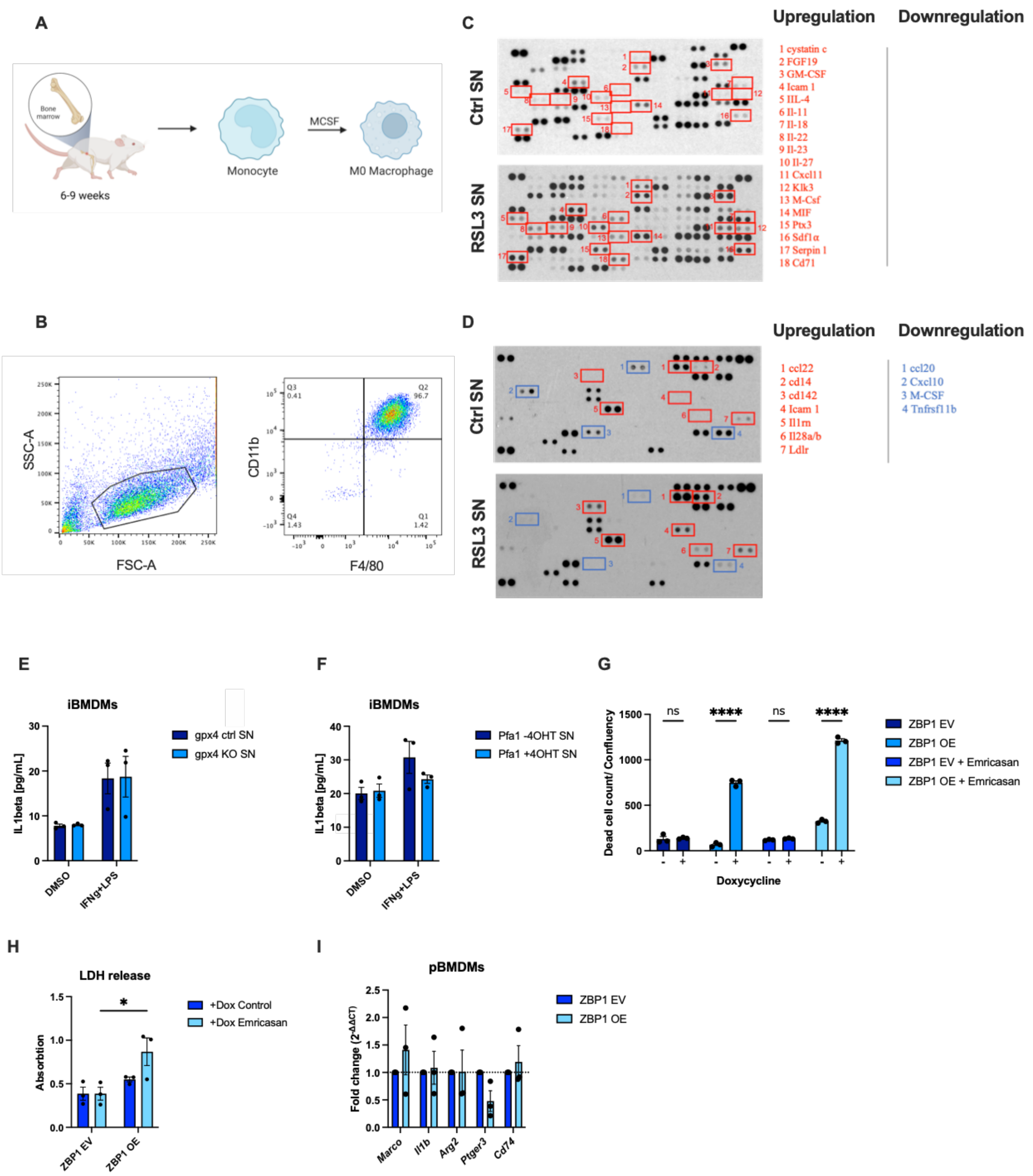

Figure S6. Ferroptotic supernatants induce limited chemo-/cytokine release from human and mouse macrophage cell lines.

(A) Schematic of pBMDMs isolation and differentiation. 6-9-week old C57BL/6N strain mice were sacrificed and bone marrow cells were isolated and differentiated for 7 days with M-CSF [25ng/ml] as described before (Toda *et al*, 2021).

(B) pBMDM differentiation at day 7 was confirmed with $CD11b^{+}$ $F4/80^{+}$ gating within live cells. Cells were analysed by flow cytometry.

(C) Pre-activated THP1 cells were incubated with Fer-1-spiked [1µM] supernatants from H441 cells treated with DMSO or RSL3 [1µM] for 24 h. Dot Blot analysis on the supernatants was performed using the cytokine profiler human XL cytokine array (R&D Systems).

(D) Raw 264.7 cells were incubated with Fer-1-spiked [1µM] supernatants from MEF cells treated with DMSO (Ctrl SN) or RSL3 [1µM] (RSL3 SN) for 24 h. Dot Blot analysis on the supernatants was performed using the cytokine profiler mouse XL cytokine array (R&D Systems).

(E) iBMDMs were incubated with SCLC ctrl. (GPX4 ctrl SN) or GPX4 KO (GPX4 KO SN) supernatants as indicated for 6 h with IFN gamma [25ng/ml], after which LPS [10ng/ml] was added for a total of 24 h. IL1β was quantified using ELISA.

(F) iBMDMs were incubated with Pfa1 ctrl. (Pfa1 -4OHT SN) or KO (Pfa1 +4OHT SN) supernatants as indicated for 6 h with IFN gamma [25ng/ml], after which LPS [10ng/ml] was added for a total of 24 h. IL1β was quantified using ELISA.

(G) Empty vector inducible (ZBP1 EV) ZBP1-inducible (ZBP1 OE) MEFs were pre-treated +/- doxycycline [1µg/ml] for 16 h and +/- Emricasan [2.5µM] for 24 h. DRAQ7 [100nM] was added to all wells to visualize dead cells. Images were acquired every 2 h using the IncuCyte SX5 bioimaging platform.

(H) ZBP1-EV and ZBP1-OE cells were treated as in (G) and subsequently were subjected to LDH quantification using a colorimetric assay.

(I) pBMDMs were incubated with supernatants from empty vector inducible (ZBP1 EV) and ZBP1-inducible (ZBP1 OE) MEFs that were pre-treated +/- doxycycline [1µg/ml] for 16 h and +/- Emricasan [2.5µM] for 24 h. The indicated transcripts were quantified by qPCR. All schemes were created with BioRender.com. Data information: (E-I) Graphs show data with means +/- SEM of at least three independent experiments. One- or two-way ANOVA was used to calculate p-values. ns: not significant; *: p<0.05; **: p<0.01; ***: p<0.001; ****: p<0.0001

A

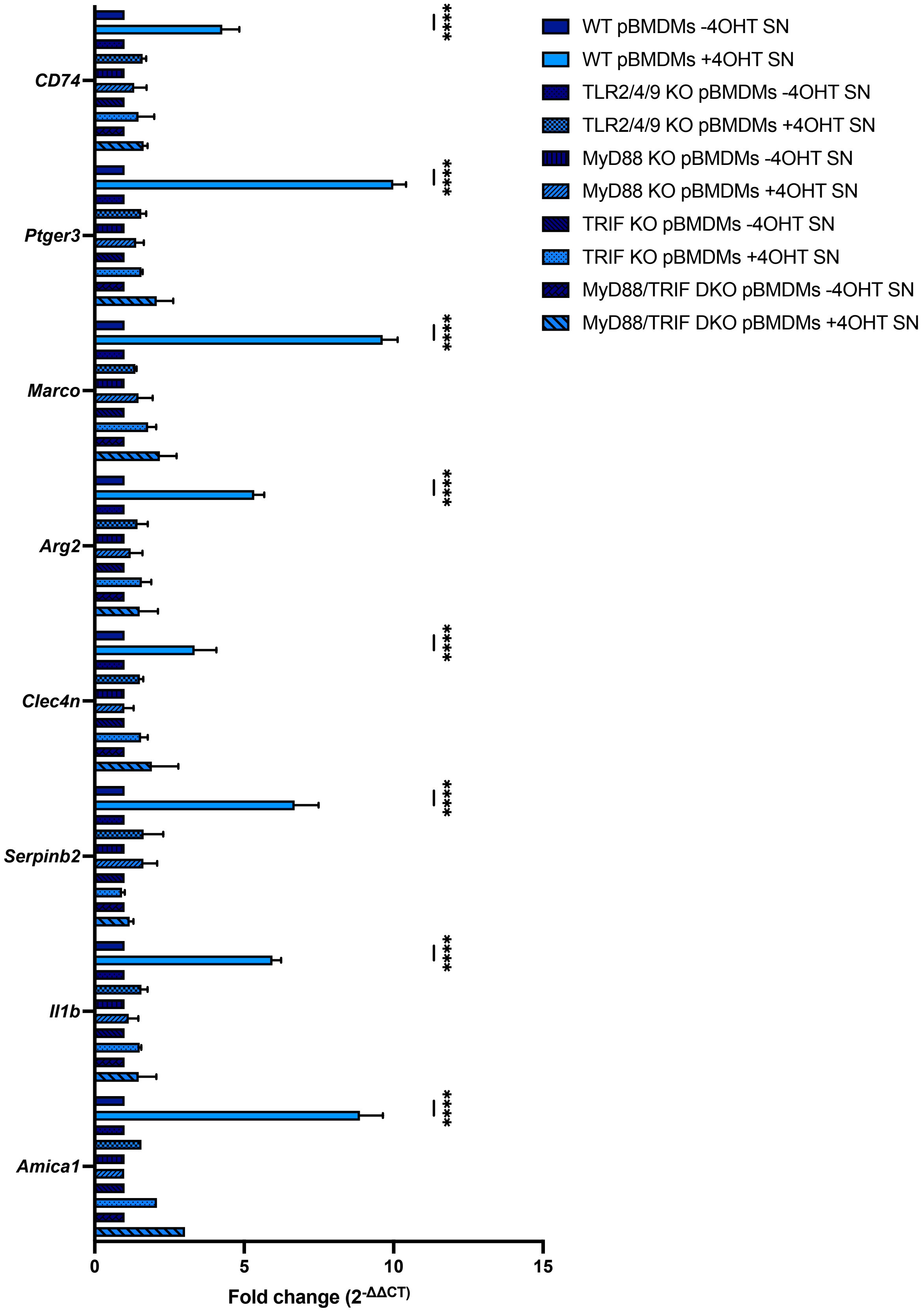

CD74
Ptger3
Marco
Arg2
Clec4n
Serpinb2
Il1b
Amica1
****
0
5
10
15
Fold change (2^-ΔΔCT)
WT pBMDMs -4OHT SN
WT pBMDMs +4OHT SN
TLR2/4/9 KO pBMDMs -4OHT SN
TLR2/4/9 KO pBMDMs +4OHT SN
MyD88 KO pBMDMs -4OHT SN
MyD88 KO pBMDMs +4OHT SN
TRIF KO pBMDMs -4OHT SN
TRIF KO pBMDMs +4OHT SN
MyD88/TRIF DKO pBMDMs -4OHT SN
MyD88/TRIF DKO pBMDMs +4OHT SN

Figure S7. Ferroptotic supernatants fail to prime pBMDMs in the absence of TLRs and adaptor proteins.

(A) Wildtype (WT), TLR2/4/9 triple knockout (KO), MyD88 KO, TRIF KO and MyD88/TRIF double KO pBMDMs were subjected to the indicated supernatants from Pfa1 MEFs for 24 h. The indicated transcripts were quantified by qPCR. Data information: (A) Graphs show data of means +/- SEM of at least three independent experiments. One- or two-way ANOVA was used to calculate p-values. ns: not significant; *: $p<0.05$; **: $p<0.01$; ***: $p<0.001$; ****: $p<0.0001$

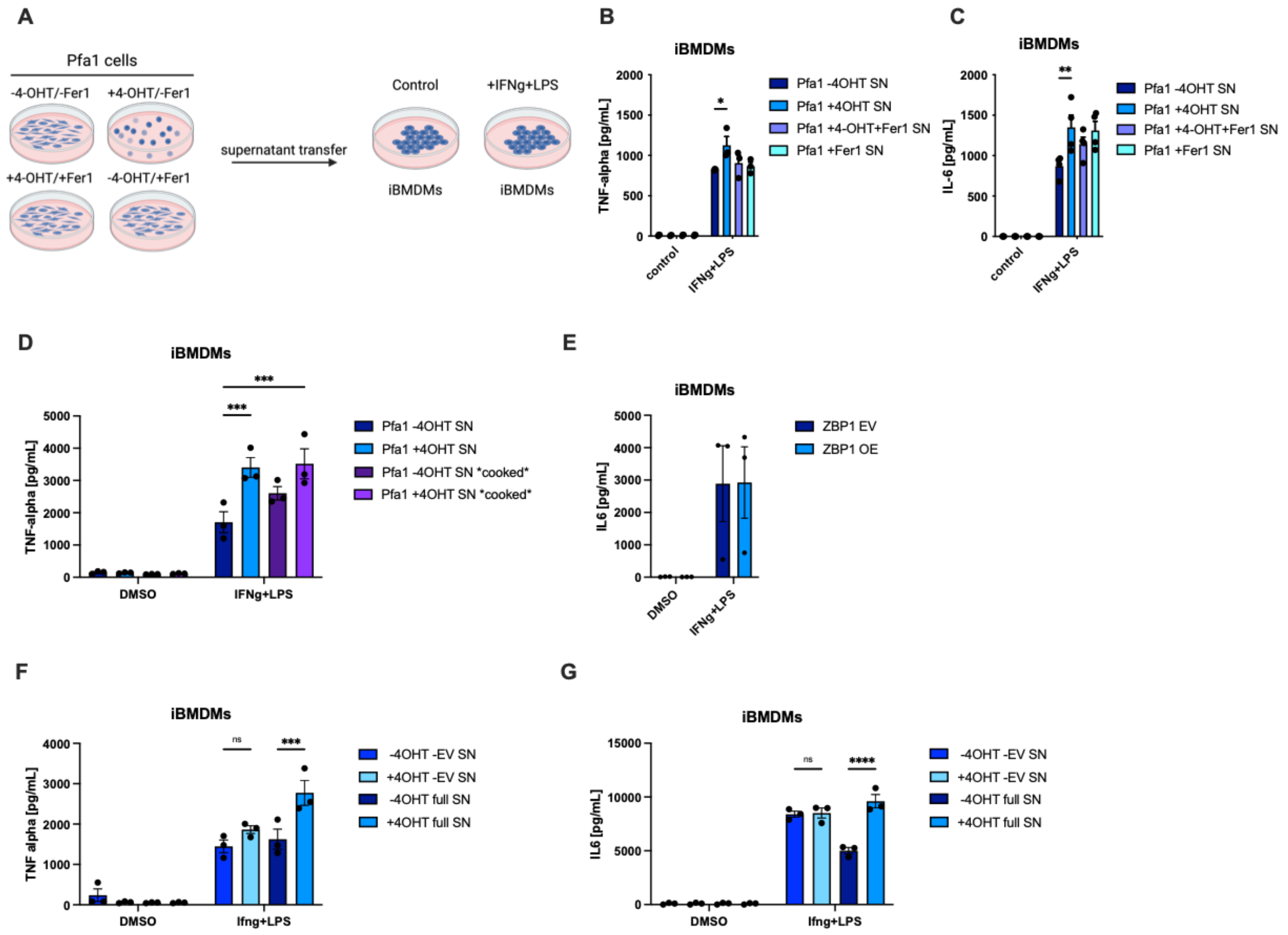

Figure S8. Ferroptotic supernatants enhance chemo-/cytokine release from iBMDMs.

(A) Schematic of supernatant transfer strategy from Pfa1 MEFs 72 h after +/- 4OHT [1µM] +/ - Ferrostatin-1 (Fer1) [1µM] treatment iBMDMs.

(B) iBMDMs were incubated with Pfa1 ctrl. (-4OHT SN), KO supernatants (+4OHT SN) +/- Ferrostatin-1 (Fer1) [1µM], respectively for 6 h with IFN gamma [25ng/ml], after which LPS [10ng/ml] was added for a total of 24 h. TNF-alpha release was quantified using ELISA.

(C) iBMDMs were incubated with Pfa1 ctrl. (-4OHT SN), KO supernatants (+4OHT SN) +/- Ferrostatin-1 (Fer1) [1µM], respectively for 6 h with IFN gamma [25ng/ml], after which LPS [10ng/ml] was added for a total of 24 h. IL-6 release was quantified using ELISA.

(D) iBMDMs were incubated with Pfa1 ctrl. (-4OHT SN), KO supernatants (+4OHT SN) or boiled "cooked" versions of supernatants for 6 h with IFN gamma [25ng/ml], after which LPS [10ng/ml] was added for a total of 24 h. TNF-alpha release was quantified using ELISA.

(E) iBMDMs were incubated with empty vector (EV) or supernatants from ZBP1-inducible (ZBP1 OE) MEFs + Emricasan [2.5 µM] for 6 h with IFN gamma [25ng/ml], after which LPS [10ng/ml] was added for a total of 24 h. IL6 release was quantified using ELISA.

(F) iBMDMs were incubated with Pfa1 ctrl. (-4OHT full SN), KO supernatants (+4OHT full SN) or EV clear (-/+ 4OHT -EV SN) versions of supernatants for 6 h with IFN gamma [25ng/ml], after which LPS [10ng/ml] was added for a total of 24 h. TNF-alpha release was quantified using ELISA.

(G) iBMDMs were incubated with Pfa1 ctrl. (-4OHT full SN), KO supernatants (+4OHT full SN) or EV clear (-/+ 4OHT -EV SN) versions of supernatants for 6 h with IFN gamma [25ng/ml], after which LPS [10ng/ml] was added for a total of 24 h. IL6 release was quantified using ELISA. All schemes were created with BioRender.com. Data information: (B-G) Graphs show data with means +/- SEM of at least three independent experiments. One- or two-way ANOVA was used to calculate p-values. ns: not significant; *: $p<0.05$; **: $p<0.01$; ***: $p<0.001$; ****: $p<0.0001$
