# Supplementary figures and images for: An atlas of ferroptosis-induced secretomes
Source: Cell Death Differ. 2025 Apr 25;32(11):1986–2008. doi: 10.1038/s41418-025-01517-4 (PMC12572367; doi:10.1038/s41418-025-01517-4)

Fig. 1L

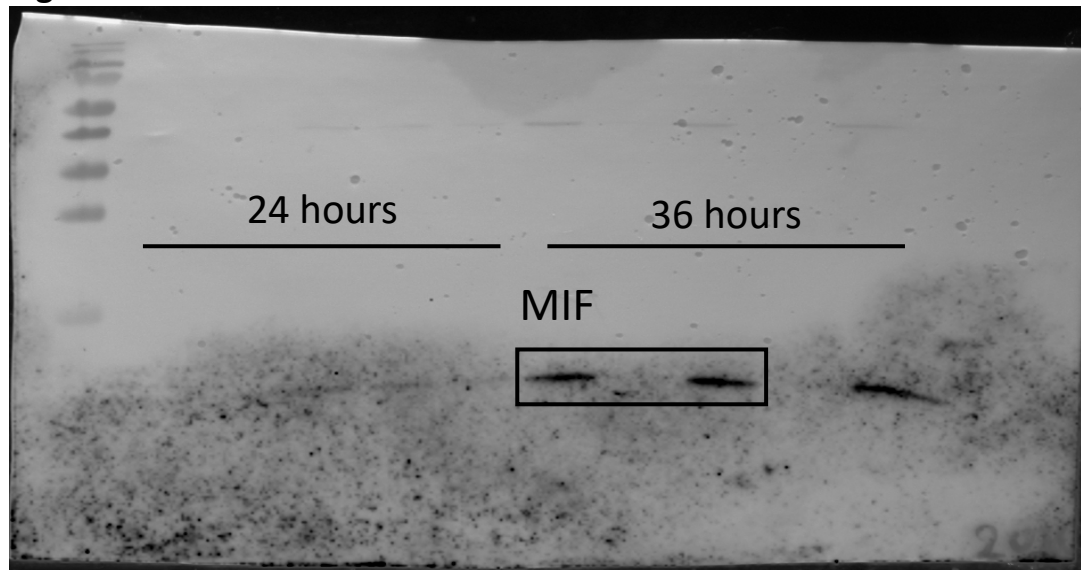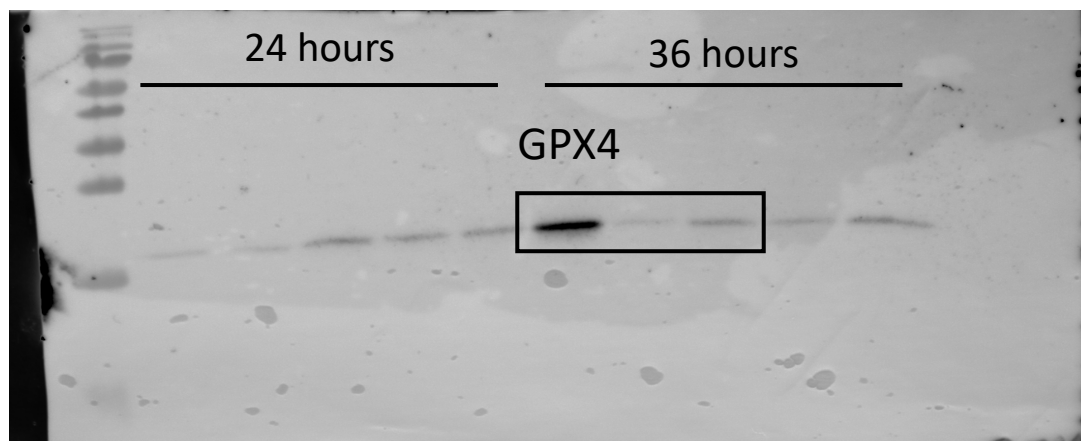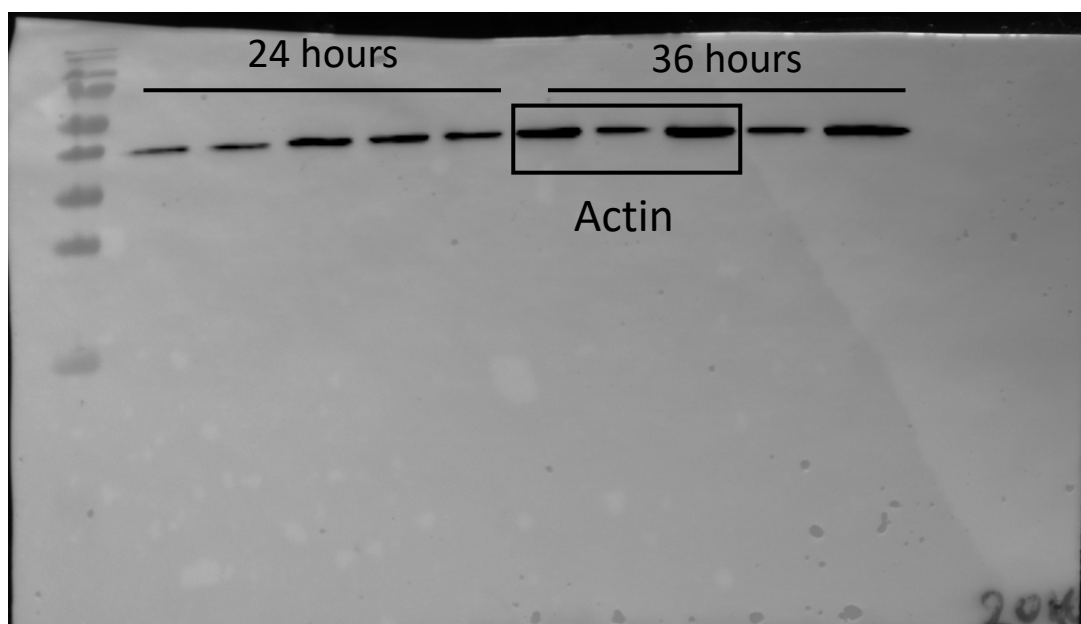

Figure S2A

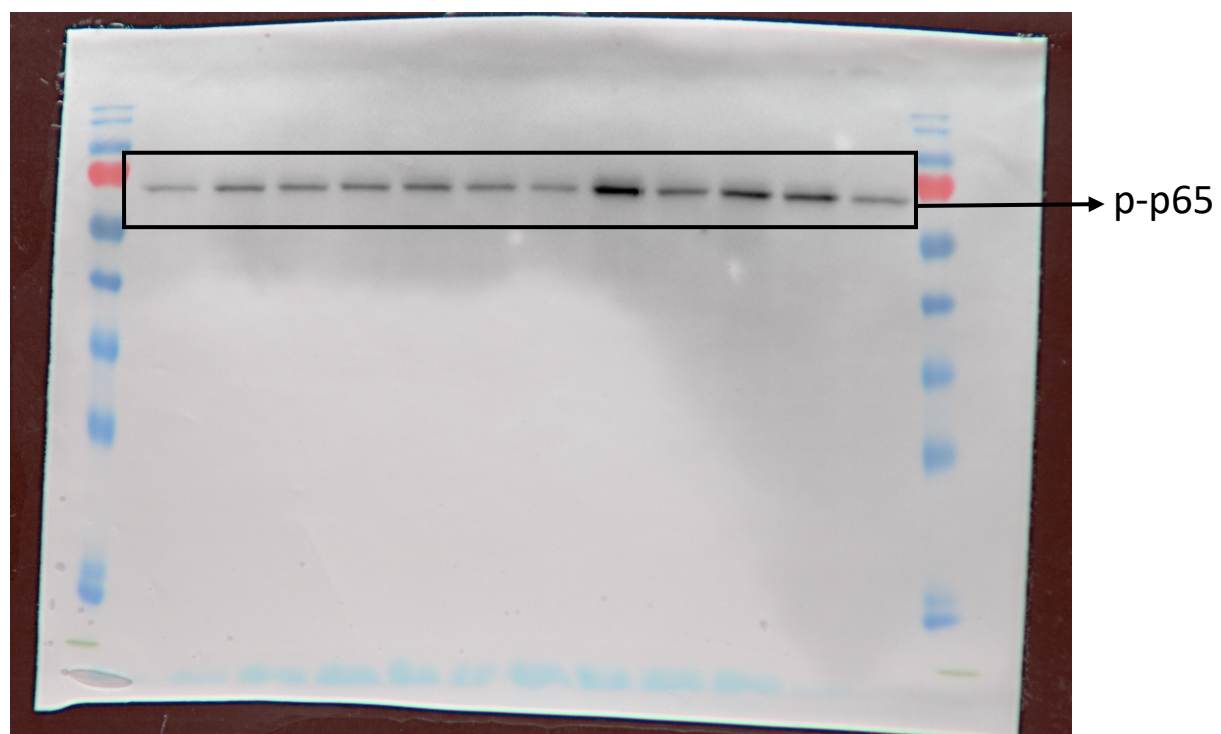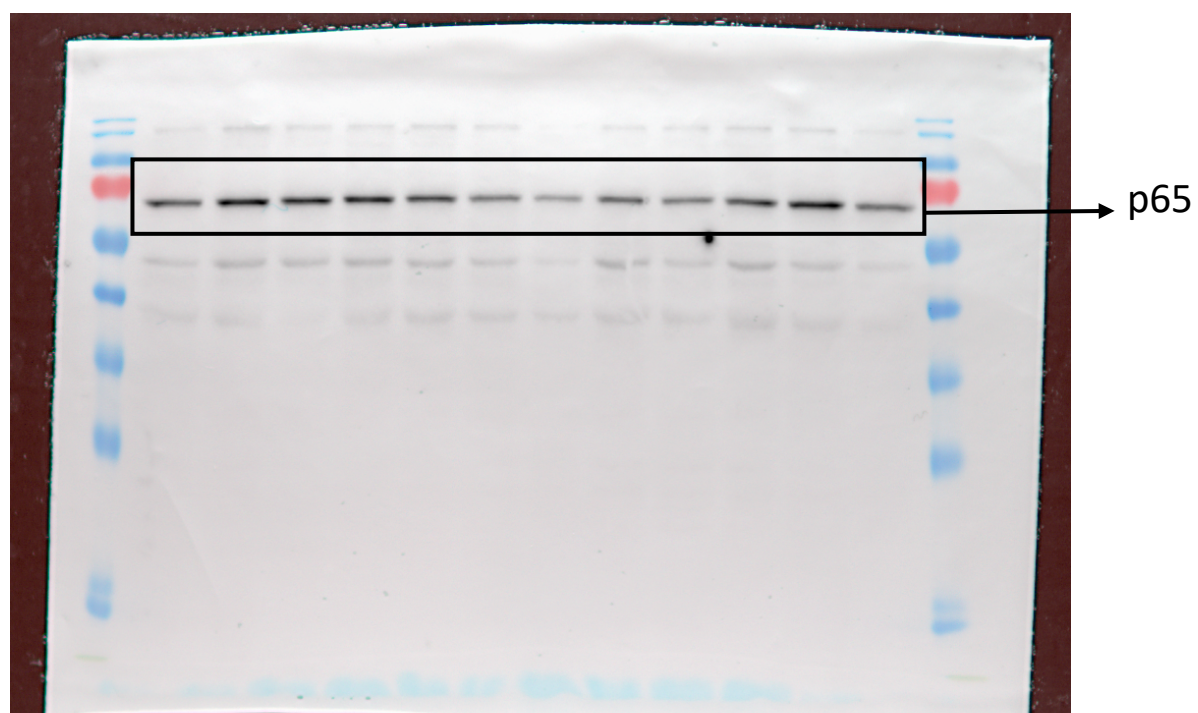

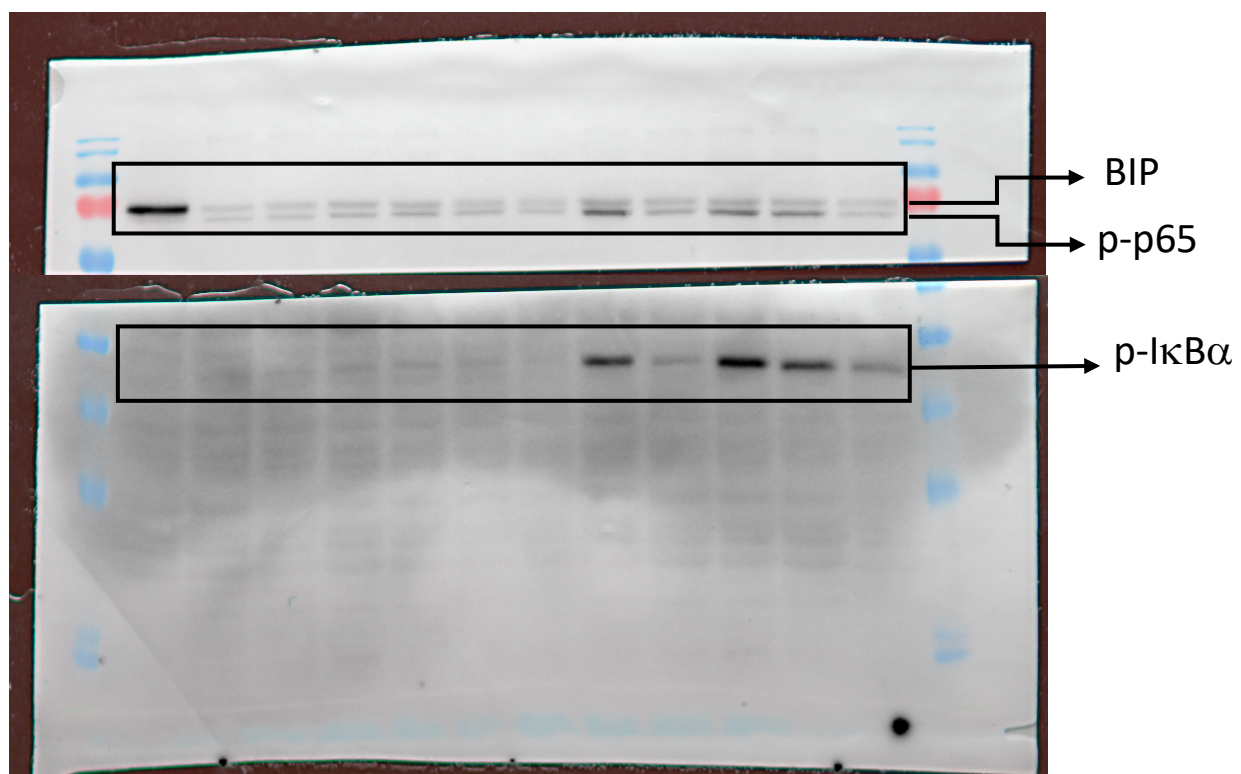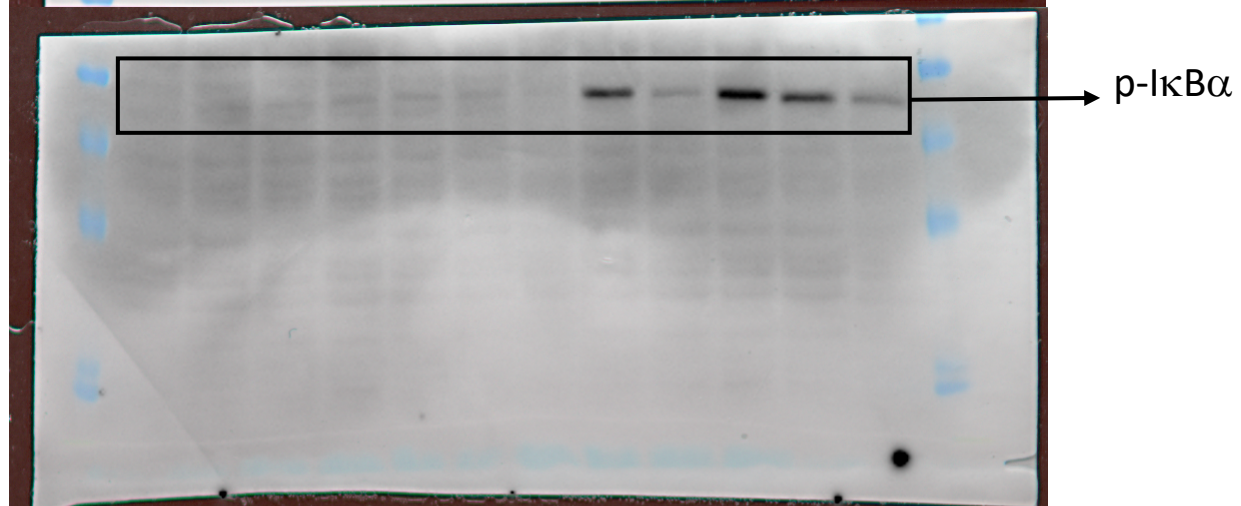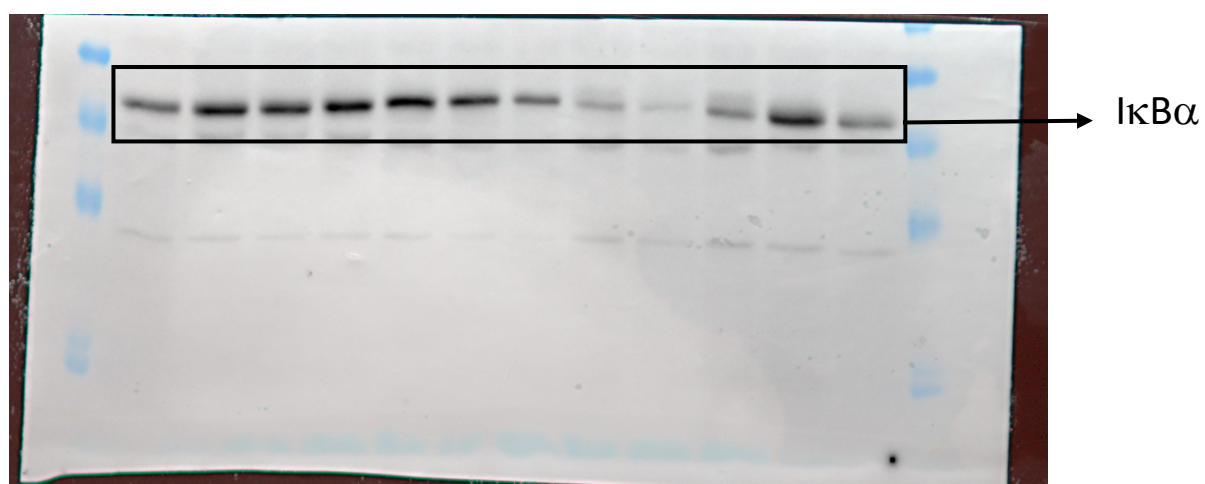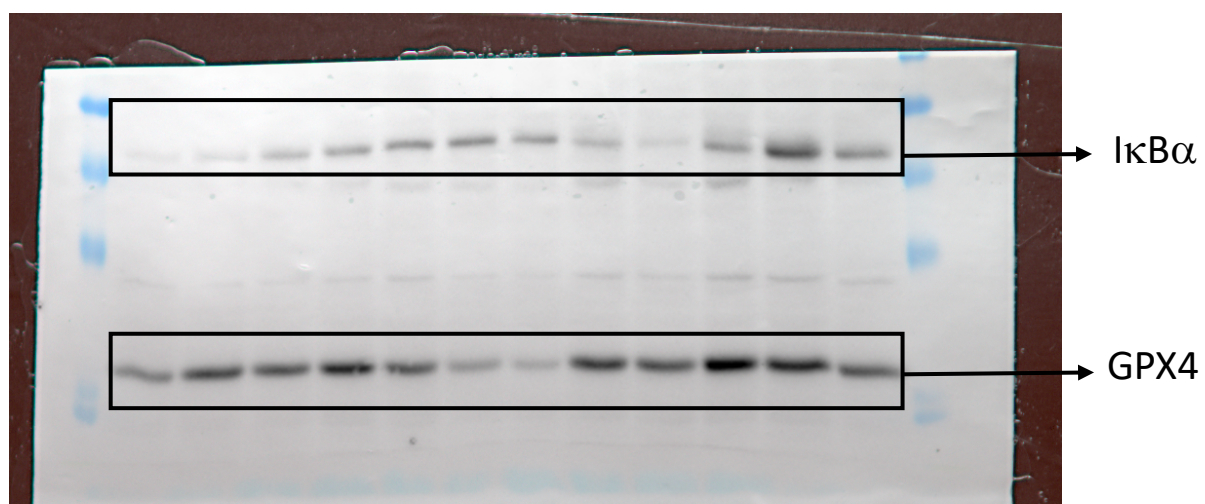

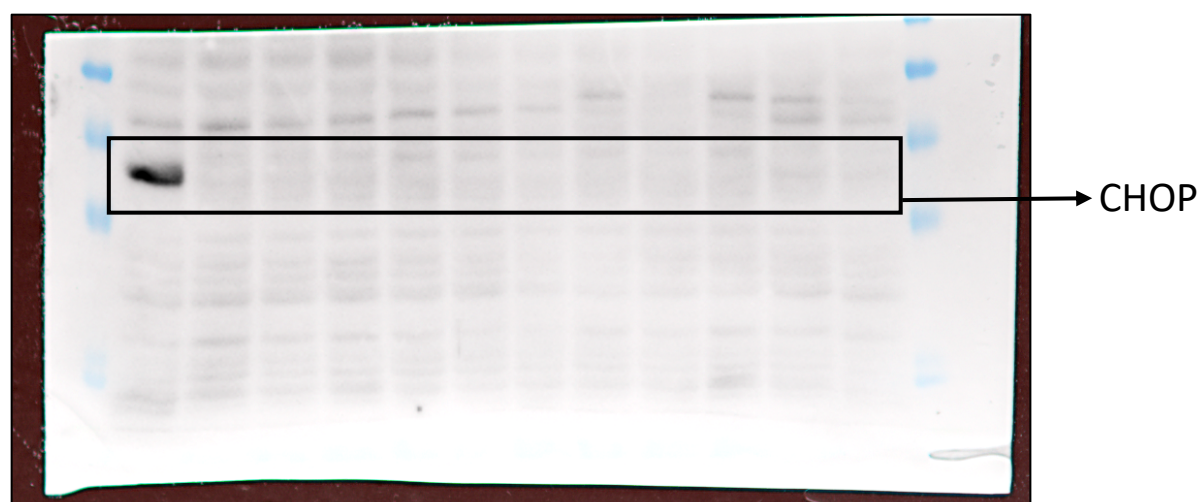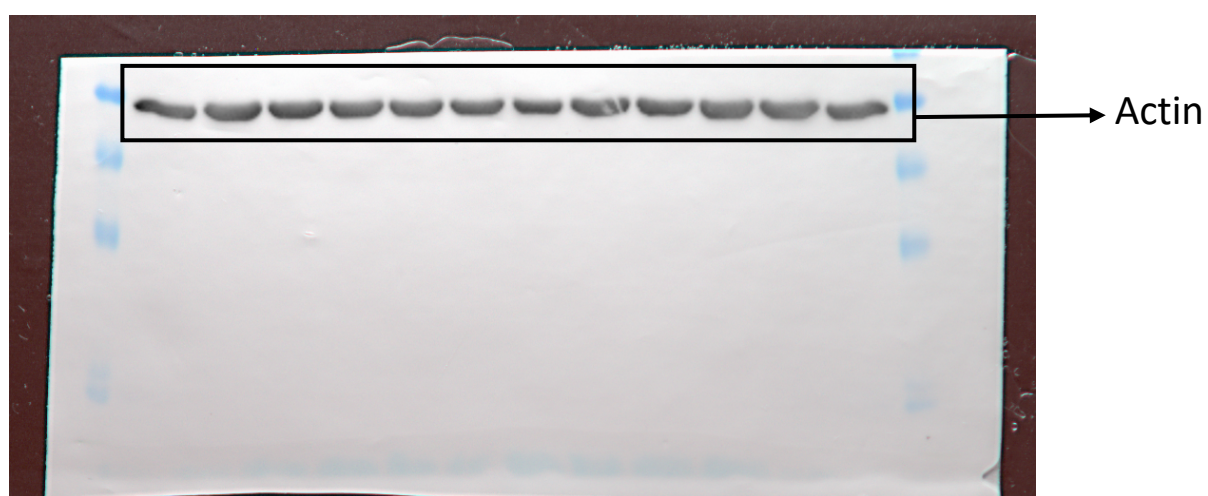

Figure S3A

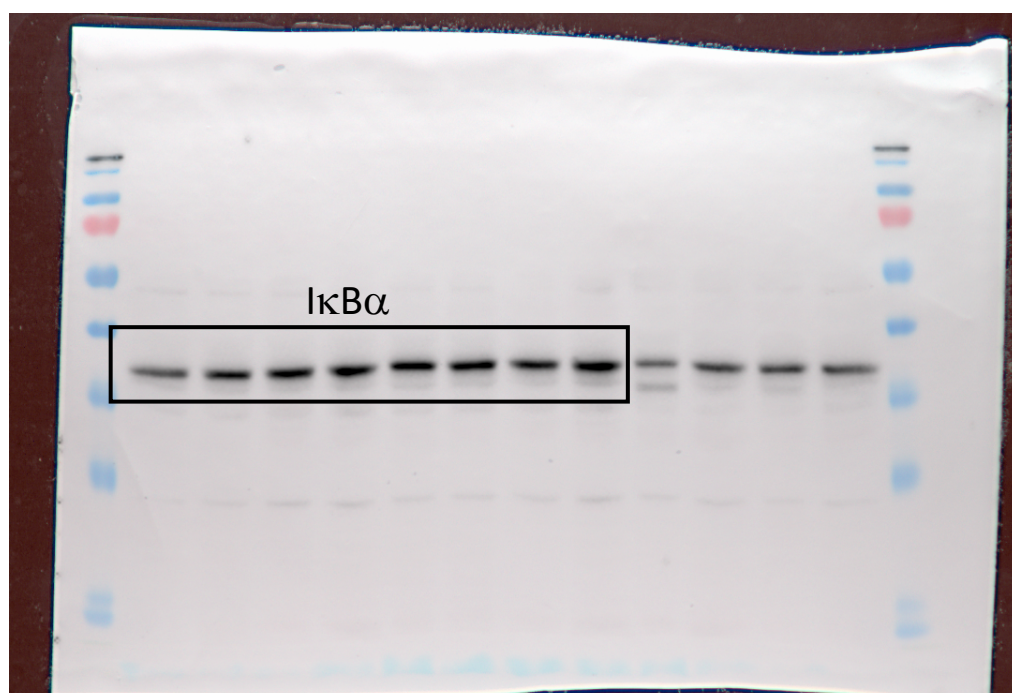

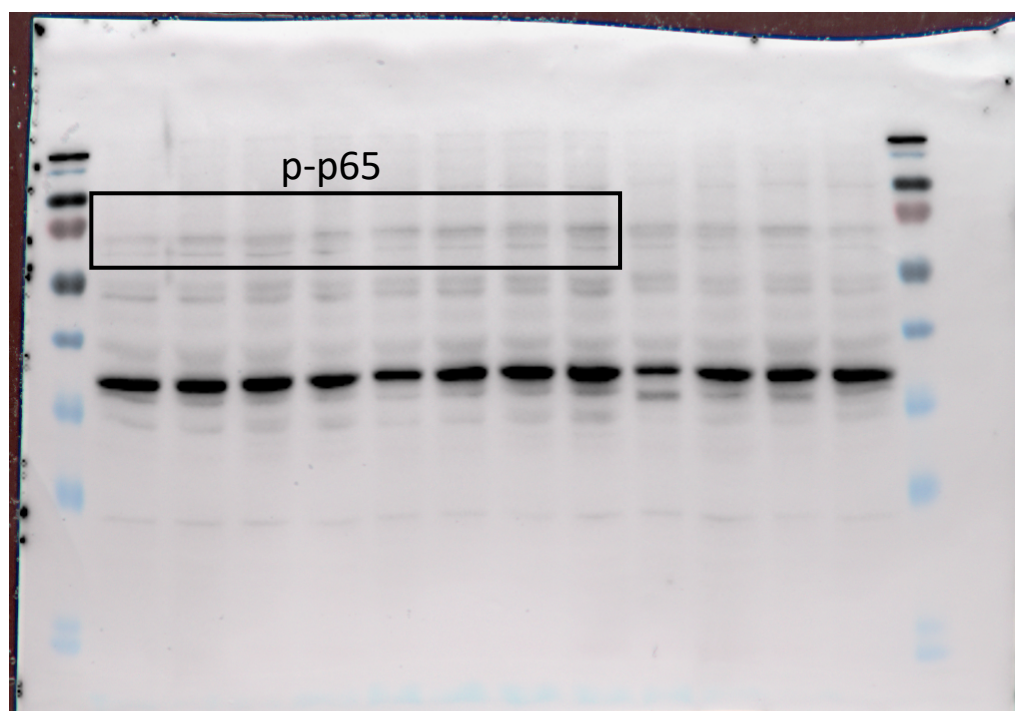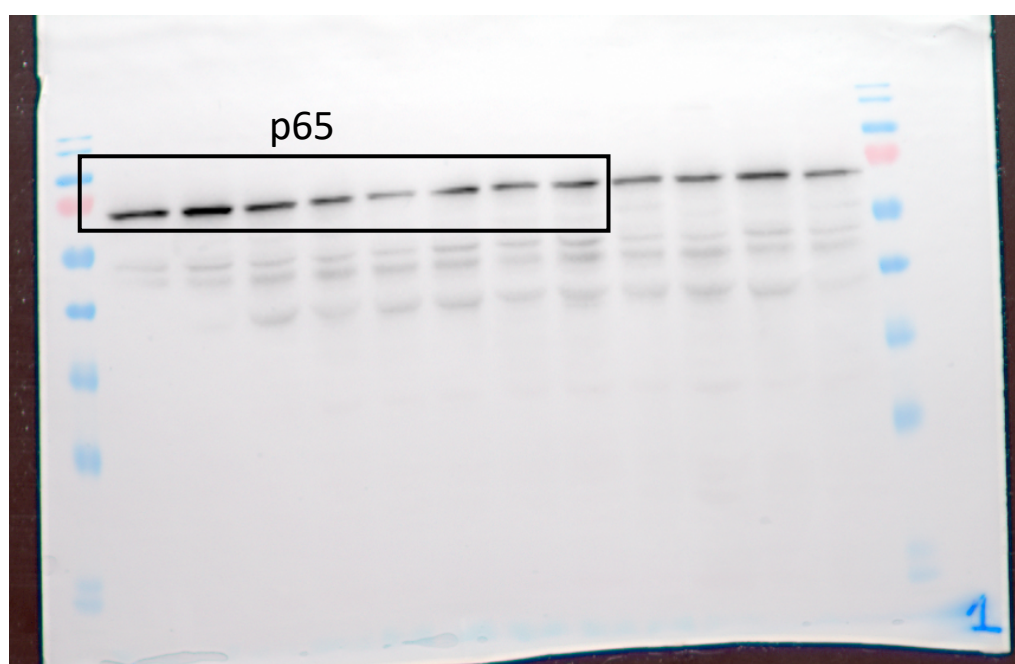

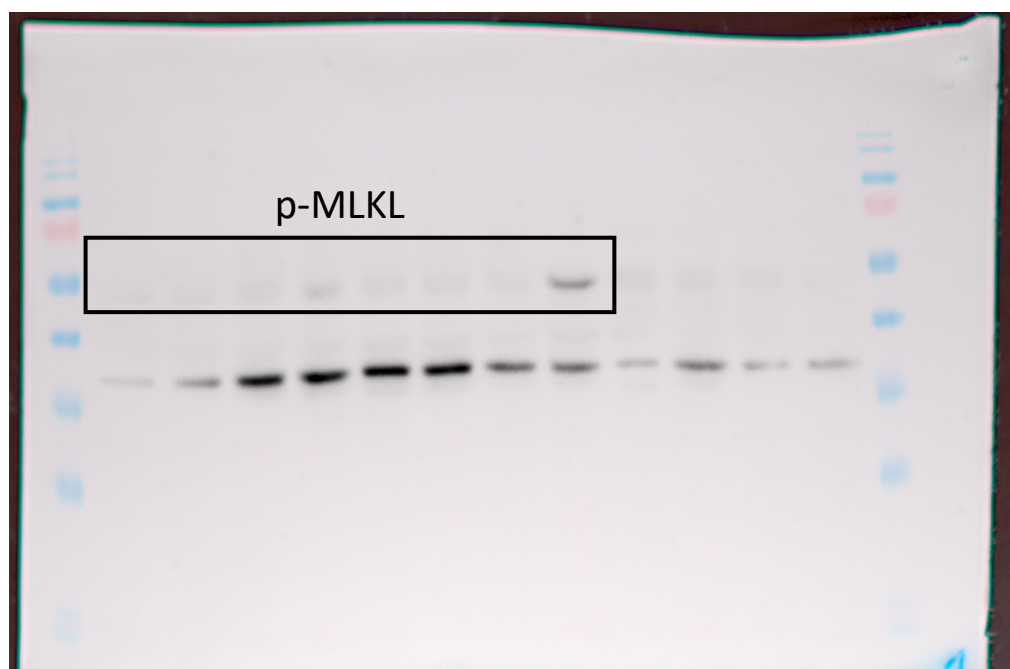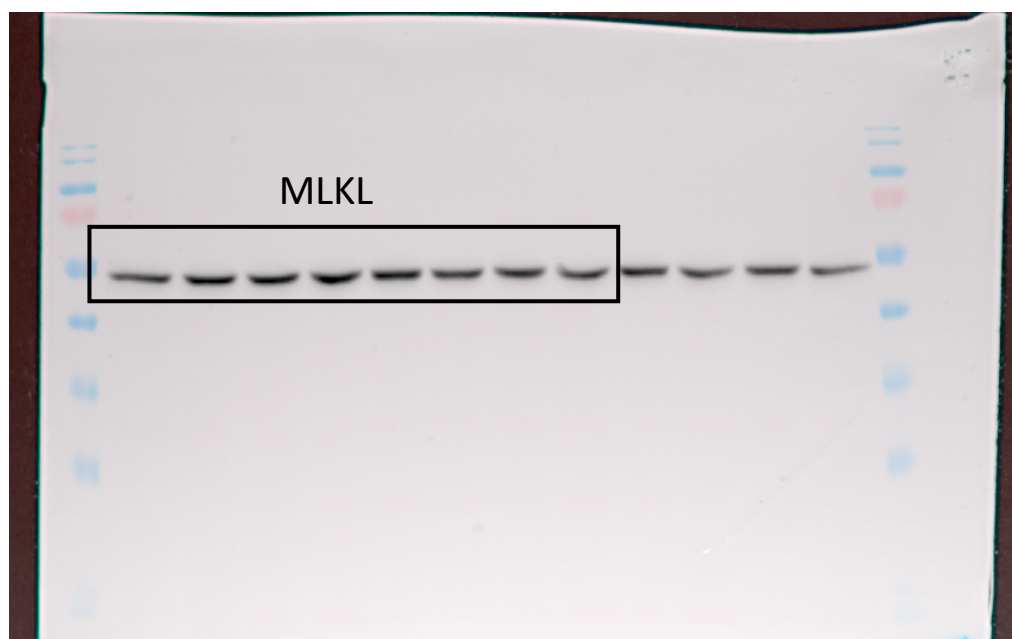

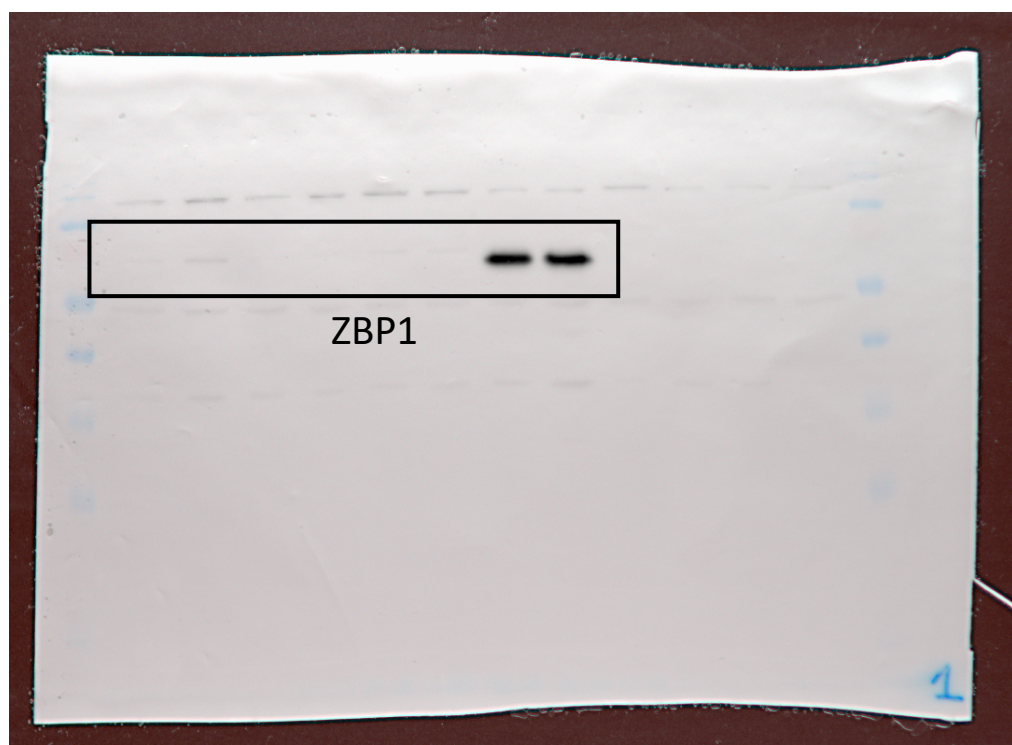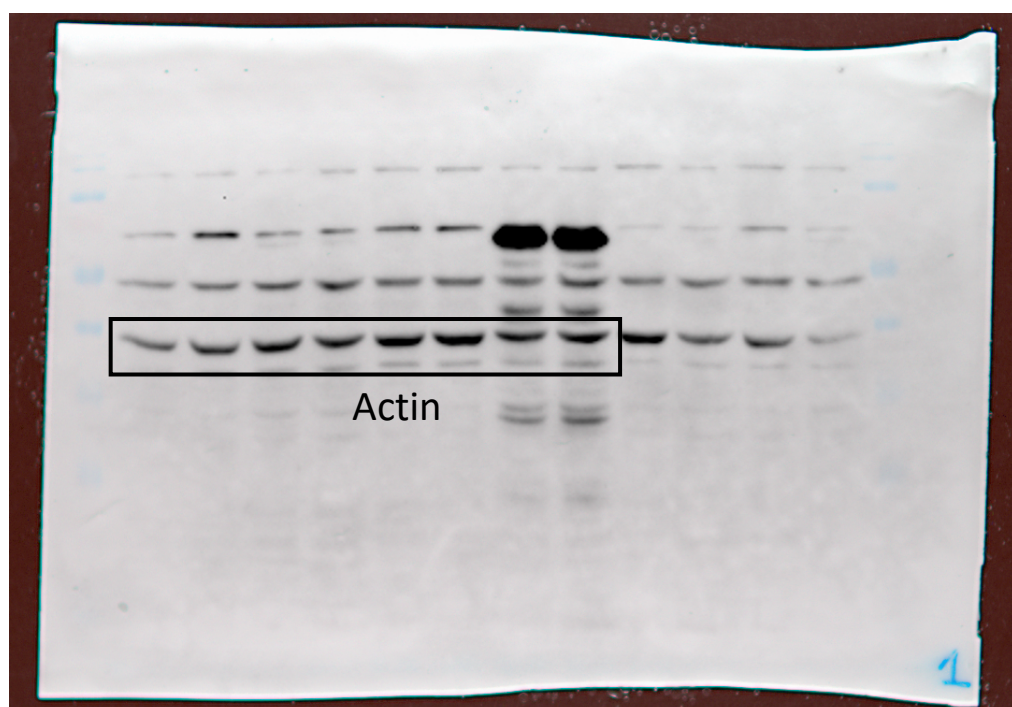

Supplement: Supplementary file 2 — Original data file Western Blots [file 41418_2025_1517_MOESM2_ESM.pdf]
